# Energy Expenditure in Upper Gastrointestinal Cancers: a Scoping Review

**DOI:** 10.1016/j.advnut.2023.08.002

**Published:** 2023-08-08

**Authors:** Lauren Hanna, Judi Porter, Judy Bauer, Kay Nguo

**Affiliations:** 1 Department of Nutrition, Dietetics and Food, Monash University, Clayton, Victoria, Australia; 2 Institute for Physical Activity and Nutrition, School of Exercise and Nutrition Sciences, Deakin University, Geelong, Victoria, Australia

**Keywords:** cancer, upper gastrointestinal cancer, energy expenditure, indirect calorimetry, doubly labeled water, scoping review, oncology, malnutrition

## Abstract

Malnutrition is prevalent in people with upper gastrointestinal (GI) cancers and is associated with shorter survival and poor quality of life. In order to effectively prevent or treat malnutrition, nutrition interventions must ensure appropriate energy provision to meet daily metabolic demands. In practice, the energy needs of people with cancer are frequently estimated from predictive equations which are not cancer-specific and are demonstrated to be inaccurate in this population. The purpose of this scoping review was to synthesize the existing evidence regarding energy expenditure in people with upper GI cancer. Three databases (Ovid MEDLINE, Embase via Ovid, CINAHL plus) were systematically searched to identify studies reporting on resting energy expenditure using indirect calorimetry and total energy expenditure using doubly labeled water (DLW) in adults with any stage of upper GI cancer at any point from diagnosis. A total of 57 original research studies involving 2,125 individuals with cancer of the esophagus, stomach, pancreas, biliary tract, or liver were eligible for inclusion. All studies used indirect calorimetry, and one study used DLW to measure energy expenditure, which was reported unadjusted in 42 studies, adjusted for body weight in 32 studies, and adjusted for fat-free mass in 13 studies. Energy expenditure in upper GI cancer was compared with noncancer controls in 19 studies and measured compared with predicted energy expenditure reported in 31 studies. There was heterogeneity in study design and in reporting of important clinical characteristics between studies. There was also substantial variation in energy expenditure between studies and within and between cancer types. Given this heterogeneity and known inaccuracies of predictive equations in patients with cancer, energy expenditure should be measured in practice wherever feasible. Additional research in cohorts defined by cancer type, stage, and treatment is needed to further characterize energy expenditure in upper GI cancer.

**Statement of Significance**

The present scoping review comprehensively and systematically synthesizes a large body of evidence to refine our understanding of energy expenditure in upper gastrointestinal cancer. This review provides a foundation for future work to improve accuracy in the prediction of daily energy requirements, which is vital for the development of effective nutrition interventions in research and clinical practice for this nutritionally vulnerable population.

## Introduction

Cancers of the upper gastrointestinal (GI) tract are among the leading causes of cancer death globally, with the number of deaths worldwide in 2020 exceeding 544,000 for esophageal cancer, 768,000 for gastric cancer, 830,000 for liver cancer, and 466,000 for pancreatic cancer [1]. The low survival rates for upper GI cancers can largely be attributed to it’s often times advanced disease stage at diagnosis [2]. Treatment options for upper GI cancer include surgical resection and/or medical therapies depending on treatment intent (curative vs. palliative), with the overall aim of treatment to optimize both length and quality of life.

Malnutrition, specifically “undernutrition” resulting from insufficient intake or metabolic uptake of nutrients [3], occurs frequently as a comorbidity of upper GI cancer [4]. The potential for reduction in food intake caused by mechanical obstruction by the tumor or side effects of treatment such as nausea and vomiting, compounded by a tumor-associated increase in metabolic demand in some cases [5, 6], contributes to the high prevalence of malnutrition in this patient group [7, 8]. Cancer-associated malnutrition increases risk of morbidity and mortality [9, 10], reduces health-related quality of life [11], and generates a significant economic burden for health services [12]. Optimization of nutrition status through effective nutrition intervention is, therefore, imperative.

Fundamentally, nutrition interventions must facilitate the meeting of patients’ daily macro and micronutrient requirements to prevent or treat malnutrition. A foundation for the provision of effective nutrition intervention in people with cancer is an accurate understanding of daily energy expenditure and daily energy requirements [13, 14]. Total energy expenditure (TEE) is comprised of resting or basal energy expenditure (approximately two-thirds), along with physical activity (20–30%) and meal-induced thermogenesis (5–10%) [15]. Doubly labeled water (DLW) is the reference method for determining TEE through measurement of dilution spaces and the elimination rates of tracers (via spot urine collection) over a period of 7 to 14 d after ingestion of water labeled with the stable isotopes deuterium and oxygen-18; the difference in elimination rates is proportional to carbon dioxide production and is used to calculate TEE [16, 17]. However, the DLW method is costly [18] and can be practically prohibitive in clinical practice [19, 20]. Indirect calorimetry is the reference method for measuring resting energy expenditure (REE) through pulmonary gas exchange [21]; however, predictive equations based on anthropometrical or demographic data in conjunction with stress or activity factors are frequently used as quicker and lower cost substitutes for the determination of TEE. Commonly used predictive equations, such as the Schofield or Harris Benedict equations, were developed through the study of healthy subjects and have been demonstrated to be inaccurate in cancer cohorts [15, [22], [23], [24]].

Basing nutrition interventions on imprecise estimations has implications for patients regardless of the direction of error; underestimation will facilitate negative energy balance and could exacerbate a decline in nutrition status [22], whereas overestimation and overfeeding confer risk of poor glycemic control, dyslipidemia, and organ dysfunction [25, 26]. For patients with upper GI cancers where risk and prevalence of malnutrition are high relative to other cancer types [27], accuracy in energy provision through nutrition intervention is of considerable importance. To enhance the understanding of energy requirements in this population, the broad aim of this scoping review was to systematically synthesize the existing evidence relating to energy expenditure in upper GI cancers. Specific aims were to i) report objectively measured energy expenditure using reference methods (REE using indirect calorimetry or TEE using DLW) and ii) summarize any reported comparisons of these measures with those of healthy (noncancer) cohorts and/or predictive equations. It was hypothesized that there would be heterogeneity in both reported measures of energy expenditure and in the agreement between these measures and comparator values.

## Methods

This scoping review is reported according to the PRISMA Extension for Scoping Reviews (PRISMA-ScR) [28]. The protocol for this scoping review was prospectively registered on Open Science Framework on 1^st^ December 2022 (https://doi.org/10.17605/OSF.IO/CX5Z9).

### Eligibility criteria

Primary research studies reporting on energy expenditure (Outcome) measured using reference methods such as indirect calorimetry or DLW (Intervention) in people with upper GI cancers (Population) were eligible for inclusion. Outcomes of interest were daily resting or TEE measures expressed as an unadjusted value (e.g., kcal/day), and/or adjusted for body weight (kcal/kg/day) or fat-free mass (FFM, e.g., kcal/kg FFM/day). Additional outcomes of interest were comparisons between measured energy expenditure in upper GI cancer and noncancer control groups and/or predictive equations, as well as details of any further subgroup analyses. For the purpose of this review, upper GI cancers were defined as cancers of the esophagus, stomach, pancreas, biliary tract, gall bladder, liver, or small intestine, according to the Australian Institute of Health and Welfare definition [29]. Studies investigating multiple cancer types were included if energy expenditure data was reported for each cancer type. Studies with summary data reported for a cohort of participants with mixed cancer types were included if all cancer types were upper GI and excluded if the cohort included any non-upper GI cancers. Letters, conference abstracts, systematic or narrative reviews, and studies not able to be translated into English using Google Translate were excluded.

### Search strategy

A systematic search of 3 databases was conducted on 29^th^ November 2022 following consultation with an experienced medical librarian to ensure a comprehensive search strategy: Ovid MEDLINE and Epub Ahead of Print, In-Process, In-Data-Review and Other NonIndexed Citations, Daily and Versions (1946 to date), Embase via Ovid (1947 to date), and CINAHL plus (EBSCOhost) (1937 to date). The search strategy included a combination of Medical Subject Headings (MeSH headings) and keywords; details of search terms used for each database are available in Supplementary File 1.

### Study selection

References identified through database searching were exported to Endnote X9 [30], where duplicates were removed by the software. The remaining references were then uploaded to Covidence [31] for independent title and abstract screening by 2 researchers. Full-text review of potentially eligible studies was conducted in duplicate. Conflicts were resolved through consensus discussion before progressing through each stage of screening.

### Data extraction

Data extraction was conducted using a customized Excel spreadsheet which had been previously piloted with studies identified in preliminary literature searches and refined through discussion between researchers (LH and KN). Data extraction was completed by one researcher (LH), with an independent review of this data shared between the remaining 3 researchers. Any errors or inconsistencies in the presentation of data that were identified through this process were corrected. Details recorded included study characteristics (country, study design, setting, sample size), participant demographics, and clinical information [age, sex, cancer type/s and stage, treatment status, weight, BMI, FFM, energy expenditure assessment methods (i.e., indirect calorimetry, DLW), and energy expenditure results (unadjusted energy expenditure per day, and/or adjusted for body weight and/or FFM)]. Comparative data were also extracted, e.g., energy expenditure predicted using an equation or ratio, or energy expenditure measured in a noncancer cohort. Data relating to predictive equations using stress factors to estimate REE was not extracted, as this was outside the scope of this review. Further details of data extracted from included studies are outlined in the registered protocol.

### Data synthesis

Included studies were grouped by cancer type when reporting on study characteristics or outcomes of interest for this scoping review. Where studies grouped participants with different upper GI cancers (e.g., “hepatobiliary” cancers [32]), duplicate data were presented in both cancer type categories. All energy expenditure data were reported in kilocalories (kcal) with the conversion from kilojoules (kJ) made where necessary using a factor based on 4.184kJ per kcal. Unadjusted energy expenditure (kcal/day) was rounded to whole numbers for reporting. In the results tables summarizing energy expenditure data, BMI data were preferentially reported where available, and weight was reported if BMI data was not available. Where mean values for variables such as energy expenditure, age, or weight were not reported, but individual participant data were reported, the mean and standard deviation of these variables was calculated by the authors. Measured REE was categorized as “hypermetabolic” if greater than 110% of predicted, “normometabolic” if between 90 and 110% of predicted, and “hypometabolic” if less than 90% of predicted, as per the method outlined by Boothby et al. [33], which was utilized in many included studies for participant classification. Due to the volume and heterogeneity of evidence identified from the database search, a narrative description and summary data are presented within this scoping review. Along with study characteristics, the number of studies reporting on energy expenditure in each cancer type, the number of studies with comparisons to noncancer controls and predictive equations, and the proportion of studies in which energy expenditure in people with upper GI cancers was found to be higher, similar, or lower than the comparator, are summarized herein. Further detail of individual studies’ sample characteristics, energy expenditure values (measured and predicted), comparisons to noncancer controls and/or predictive equations, and reference to subgroup analyses are provided in Supplementary Files 2 and 3.

## Results

### Study selection

**FIGURE 1:**
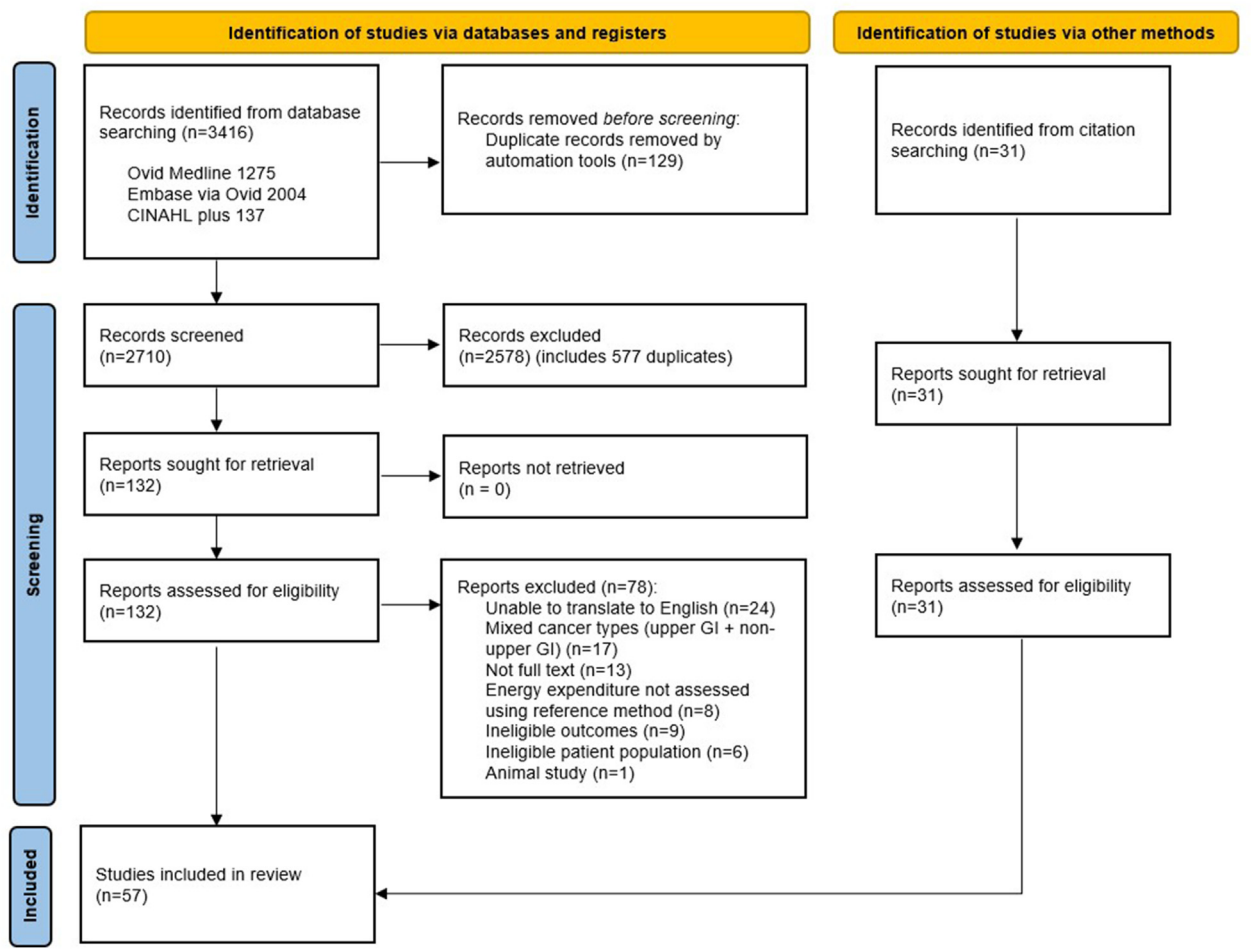
PRISMA flow diagram of study selection

The PRISMA flow diagram of study selection is presented in Figure 1. The systematic database searches yielded a total of 3,416 studies. Following the automated removal of duplicates, 2,710 references underwent title and abstract screening. Full-text review of 129 studies was conducted, with 74 studies excluded at this stage (Figure 1). A hand search of reference lists of 54 eligible studies identified 31 additional studies for screening; full-text screening of these studies was conducted in duplicate, with 3 studies found to meet eligibility criteria. A total of 57 studies were included in this scoping review.

### Study characteristics

A summary of the characteristics of the 57 included studies are presented in **Table 1** [[6], [32], [34], [35], [36], [37], [38], [39], [40], [41], [42], [43], [44], [45], [46], [47], [48], [49], [50], [51], [52], [53], [54], [55], [56], [57], [58], [59], [60], [61], [62], [63], [64], [65], [66], [67], [68], [69], [70], [71], [72], [73], [74], [75], [76], [77], [78], [79], [80], [81], [82], [83], [84], [85], [86], [87], [88]]. All 57 studies reported on REE measurements using indirect calorimetry, and only one study reported on TEE measured using DLW [34]. Studies were published between 1956 and 2022 in Japan (*n =* 20, 35.1%), UK (*n =* 10, 17.5%), China (*n =* 7, 12.3%), USA (*n =* 4, 7.0%), Brazil (*n =* 3, 5.3%), Italy (*n =* 3, 5.3%), South Africa, Scotland (both *n =* 2, 3.5%), Netherlands, France, Portugal, Australia, Korea, and Israel (all *n =* 1, 1.8%). Twenty-two studies (38%) were published between 1980 and 2000, 12 studies (21%) were published between 2001 and 2010, and 23 studies (40%) were published after 2010.

The upper GI cancer type most frequently studied was esophageal (25 studies), followed by pancreatic (19 studies), gastric (18 studies), liver (11 studies), and bile duct cancer (4 studies) (Table 1). Nine studies examined more than one upper GI cancer type [6, 32, [35], [36], [37], [38], [39], [40], [41]]. No studies of individuals with cancer of the small intestine were retrieved. One study included 9 participants with “hepatobiliary” cancer (defined as cancer of the liver, bile ducts, and/or gall bladder) [90], but as there was no explicit reference to gall bladder cancer, data from this study was categorized with liver and bile duct cancers. The cancer stage of participants was reported with varying levels of detail between studies; participants had early-stage/resectable disease in 8 studies (14%), advanced/unresectable disease in 7 studies (12%), and a mix of early and advanced disease in 23 studies (40%). In 3 studies (5%), the stage of cancer was reported for a wider cohort of participants with mixed cancers but not reported by individual cancer type. In 14 studies (24%), the stage of cancer was not reported.

**TABLE 1:** Characteristics of included studies

Author, y	Country	Study design	Sample size (*n*)^1^	Cancer type/s	Cancer stage I/II/III/IV	Cancer treatment status at time of EE assessment	Age, y (mean ± SD)	Sex Female, *n* (%)	EE assessment method
Adachi et al*.* (2010) [69]	Japan	RCT	20	Gastric	Intervention group 8/2/0/0 Control group 10/0/0/0	Post gastrectomy Other treatment not reported	Intervention (*n =* 10) 64.8 (±10.4) Control (*n =* 10) 61.6 (±8.4)	3 (30) 6 (60)	Indirect calorimeter, Med-Gem metabolic analyser, HealtheTech Inc
Barber et al. (1999) [58]	UK	Noncontrolled trial	20	Pancreatic	0/8/3/9	No prior chemo/radiotherapy No surgery within past 4 wk	62 (51–75)^2^	10 (50)	Indirect calorimeter, Deltatrac II, Datex
Barber et al. (2000) [57]	UK	Cross-sectional, then noncontrolled trial	16^3^	Pancreatic	“Unresectable”	No prior chemo/radiotherapy No surgery within past 4 wk	63 (55, 66)^4^	9 (56)^3^	Indirect calorimeter, Deltatrac, Datex
Barber et al. (2004) [72]	UK	Cross-sectional	7	Pancreatic	Not reported	No surgery or chemo/radiotherapy within past 4 wk	59 (56–75)^2^	4 (57)	Indirect calorimeter, Deltatrac II, Datex
Barcellos et al. (2021) [35]	Portugal	Cross-sectional	49	Gastric (*n =* 14) Bile duct (*n =* 17) Pancreatic (*n =* 8) Liver (*n =* 10)	“Advanced”	No prior surgery, chemotherapy, or other clinical interventions	Not reported by cancer type	Not reported by cancer type	Indirect calorimeter, Cosmed k4 b2®, Cosmed
Bauer et al. (2004) [78]	Australia	Cross-sectional	8	Pancreatic	0/3/0/5	Receiving palliative treatment, details not reported	62.0 (±5.2)	3 (38)	Indirect calorimeter, Vmax 229, Sensormedics
Becker Veronese et al. (2013) [82]	Brazil	Cross-sectional	30	Esophageal	1/10/12/7	No prior surgery or chemo/radiotherapy	61.4 (±8.6)	9 (30)	Indirect calorimeter, MetaLyzer® 3B, CORTEX Biophysik
Boudou-Rouquette et al. (2022) [36]	France	Cross-sectional	13	Gastro-esophageal (*n =* 4) Pancreatic (*n =* 9)	Not reported by cancer type	No prior cancer treatment Planned to commence chemotherapy	Not reported by cancer type	Not reported by cancer type	Indirect calorimeter, Fitmate VM®, COSMED
Cao et al. (2010) [6]	China	Cross-sectional	432	Esophageal (*n =* 150) Gastric (*n =* 154) Pancreatic (*n =* 128)	Not reported by cancer type	No prior chemo/radiotherapy Surgical intervention not reported	O: 55.3 (±7.4) G: 55.3 (±8.5) P: 57.4 (±11.2)	O: 50 (33) G: 51 (33) P: 46 (36)	Indirect calorimeter, Deltatrac Metabolic Monitor 200, Datex
Chen et al. (1994) [71]	China	Cross-sectional	57	Liver	Grouped by tumor size Small (<5cm^3^) *n =* 19 Medium (5–10cm^3^) *n =* 18 Large (>10cm^3^) *n =* 20	Prior to surgical intervention No further treatment details reported	56.4 (±1.6)	0 (0)	Indirect calorimeter, MMC Horizontal System, Beckman Instrument Inc
Chinda et al. (2017) [49]	Japan	Before-after	52	Gastric	“Early gastric cance”	Prior to ESD procedure No previous treatment	70.2 (±8.1)	13 (25)	Indirect calorimeter, METAVINE-N VMB-002N, Vine
De Jong et al. (2005) [76]	UK	Cross-sectional	15	Pancreatic	1/2/4/9	Prior to surgical intervention No previous chemo/radiotherapy	66 (±2)	6 (40)	Indirect calorimeter, Deltatrac I, S&W Vickers
Dempsey et al. (1984) [32]	USA	Cross-sectional	91	Esophageal (*n =* 26) Gastric (*n =* 28) Pancreatic (28) Hepatobiliary (*n =* 9)	Not reported by cancer type	No surgery within previous 5 d (prior surgery not reported) Not currently receiving chemo/radiotherapy treatment (prior treatment not reported)	Not reported by cancer type	Not reported by cancer type	Indirect calorimeter, Metabolic Measurement Cart, Beckman Instruments
Falconer et al. (1994) [59]	UK	Cross-sectional	21	Pancreatic	0/7/8/6	Surgical intervention in some patients > 1 mo prior (gastric bypass *n =* 10) No prior chemo/radiotherapy	57 (SEM 2)	7 (33)	Indirect calorimeter, Deltatrac Metabolic Monitor
Guglielmi et al. (1992) [60]	Italy	Cross-sectional	13	Liver	Not reported	Not reported	Well-nourished (*n =* 8) 65 (±8) Malnourished (*n =* 5) 55 (±12)	0 (0) 0 (0)	Indirect calorimeter, MMC Horizon, Sensormedics
Haffejee et al. (1985) [85]	South Africa	Noncontrolled trial	15	Esophageal	5 patients with liver metastases No other staging data	No previous surgical intervention Other treatment not reported	54.4 (range 41–79)	2 (13)	Indirect calorimeter, name of equipment not reported
Hansell et al. (1986) [65]	Scotland	Cross-sectional	22	Gastric	5 patients with liver metastases No other staging data	No surgical intervention in the previous year Other treatment not reported	66.9 (SEM 2.1)	8 (36)	Indirect calorimeter, name of equipment not reported
Hansell et al. (1987) [67]	Scotland	RCT	30	Gastric	7 patients with liver metastases No other staging data	Prior to surgical intervention Other treatment not reported	Intervention arm 1 (*n =* 10) 70.0 (SEM 2.7) Intervention arm 2 (*n =* 10) 63.3 (SEM 3.2) Control group (*n =* 10) 64.5 (SEM 3.4)	4 (40) 2 (20) 4 (40)	Indirect calorimeter, name of equipment not reported
Henz et al. (2021) [73]	Brazil	Cross-sectional	33	Liver	BCLC 0/A/B/C/D 4/12/10/4/3	Not reported	62.8 (±8.1)	7 (21)	Indirect calorimeter, MetaCheck, Korr
Hioki et al. (1990) [37]	Japan	Before-after	20	Esophageal (*n =* 10) Gastric (*n =* 10)	Not reported	Pre/-post surgical intervention (resection) Other treatment not reported	O: 58 (±8) G: 60 (±8)	1 (10) 1 (10)	Indirect calorimeter, Deltatrac, Datex
Ishikawa et al. (2004) [44]	Japan	Before-after	38	Gastric	Not reported	Presurgical/postsurgical intervention (resection) Other treatment not reported	Not reported by cancer type	Not reported by cancer type	Indirect calorimeter, Vmax29, SensorMedics
Khan et al. (2003) [45]	UK	Noncontrolled trial	10	Esophageal	“Inoperable” cancers	All had prior relief of obstructive symptoms with either palliative laser treatment or stent insertion No surgical intervention *n =* 1 received chemotherapy during trial	72.8 (range 62–86)	3 (30)	Indirect calorimeter, name of equipment not reported
Kim et al. (2013) [42]	Korea	Case study	1	Pancreatic	Not reported	Longitudinal assessments pre and post concurrent chemo-radiotherapy and total pancreatectomy	42	0 (0)	Indirect calorimeter, TrueOne2400, Parvo Medics
Klein et al. (1990) [77]	USA	Cross-sectional	5	Esophageal	Nonmetastatic	No cancer treatment prior to study	58 (SEM 2)	1 (20)	Indirect calorimeter, Horizon metabolic measurement cart, Sensormedics Corp
Kobayashi et al. (2013) [46]	Japan	Noncontrolled trial	25	Esophageal	Not reported	Studied during esophagectomy: 2-field (Group A, *n =* 10) or 3-field (Group B, *n =* 15) lymph node dissection surgery Other treatment not reported	Group A 72 (SD not reported) Group B 67 (SD not reported)	Group A 1 (10) Group B 6 (40)	Indirect calorimeter, Deltatrac, Datex
Kudo et al. (2022) [50]	Japan	Before-after	75	Esophageal	Not reported	Pre-post ESD procedure Other treatment not reported	66 (45, 90)^[^^4^^]^	8 (11)	Indirect calorimeter, METAVINE-N VMB-002N, VINE
Legaspi et al. (1987) [38]	USA	Cross-sectional	4	Esophageal (*n =* 3) Gastric (*n =* 1)	O: Nonmetastatic, *n =* 2 involving lymph nodes, *n =* 1 not involving lymph nodes G: Lymph node involvement, metastatic spread unknown	No cancer treatment prior to study	O: 55 (±14) G: 58 (N/A)	1 (33) 1 (100)	Indirect calorimeter, 1100 Medical Gas Analyzer, Perkin-Elmer
Liu et al. (2012) [51]	China	RCT	64	Gastric	Not reported	Pre-/post radical distal gastrectomy Other treatment not reported	Intervention group (*n =* 32) 59.1 (±10.1) Control group (*n =* 32) 58.9 (±11.0)	8 (25) 8 (25)	Indirect calorimeter, calcium citrate malate (CCM) nutrition metabolism investigation system, Medical Graphics Corporation
Merli et al. (1992) [63]	Italy	Cross-sectional	12	Liver	4/6/2/0	No cancer treatment at time of study Details of prior treatment not reported	64 (±3)	6 (50)	Indirect calorimeter, MMC Horizon, SensorMedics
Mitamura et al. (2011) [47]	Japan	Cross-sectional	13	Esophageal	2/4/5/2	No cancer treatment prior to study	66.5 (±8.9)	3 (23)	Indirect calorimeter (portable), VO2000, Medical Graphics Corporation
Moses et al. (2004) [34]	UK	RCT	24	Pancreatic	0/15/4/5	No surgery or chemo/radiotherapy within the previous 4 wk	68 (SEM 2)	14 (58)	REE: Indirect calorimeter, Deltatrac, Datex TEE: Doubly-labeled water, multipoint
Okamoto et al. (2001) [68]	Japan	Before-after	8	Esophageal	1/2/4/1	Pre-post oesophagectomy Other treatment not reported	58.4 (±4.3)	0 (0)	Indirect calorimeter, AE-300S, Minato Medical Science Co (Osaka, Japan)
Omagari et al. (2012) [39]	Japan	Cross-sectional	69	Esophageal (*n =* 7) Gastric (*n =* 20) Liver (*n =* 37) Pancreatic/ Biliary tract (*n =* 5)	O: 0/2/3/2 G: 0/3/1/16 H: 9/11/13/4 P/B: 0/0/0/5	Not reported	O: 70 (57–75) [2] G: 61.5 (25–79) [2] H: 70 (43–85) [2] P/B: 60 (56–79) [2]	1 (14) 10 (50) 10 (27) 2 (40)	Indirect calorimeter, Vmax SPECTRA 29n calorimeter, Cardinal Health 207 Inc
Rabito et al. (2013) [40]	Brazil	Cross-sectional	24	Esophageal or Gastric (*n =* 24)	“Palliative or curative”	Prior to surgical intervention Other treatment not reported	57 (±15)	4 (17)	Indirect calorimeter, Vmax 29, SensorMedics
Ren et al. (2019) [74]	China	Cross-sectional	80	Liver	BCLC A/B/C/D 33/13/21/13	Not reported	52.0 (±8.4)	7 (9)	Indirect calorimeter, CCM/D metabolism cart, MGC Diagnostics Corp
Saito et al. (2013) [86]	Japan	Prospective cohort	100	Liver	BCLC A/B/C/D 1-y mortality group 0/0/19/3/0 1-y survival group 9/34/34/1/0	Studied post transcatheter arterial chemoembolization (TACE) *n =* 38 had “past treatments other than TACE,” details not reported	1–y mortality group (*n =* 22) 74 (56–86) 2 1-y survival group (*n =* 78) 71 (41–87) 2	10 (45) 29 (78)	Indirect calorimeter, Deltatrac II Metabolic Monitor, Datex
Saito et al. (2014) [87]	Japan	Prospective cohort	40	Liver	Intervention group 4/7/1/1 Control group (*n =* 27) 14/10/3 (value missing)	Pre- and post-radiofrequency ablation Other treatment not reported	Intervention group (*n =* 13) 73.4 (±2.2) Control group 70.0 (±1.9)	5 (38) 11 (41)	Indirect calorimeter, Deltatrac II Metabolic Monitor, Datex
Sane et al. (2000) [52]	Japan	RCT	18	Esophageal	Intervention group 3/1/5/0 Control group 3/1/2/3	Post oesophagectomy Other treatment not reported	Intervention group (*n =* 9)^5^ 60.7 (±9.6) Control group (*n =* 9) 5 63.3 (±6.2)	0 (0) 1 (11)	Indirect calorimeter, Deltatrac, Datex
Satoh et al. (2018) [80]	Japan	Nonrandomized controlled trial	32	Esophageal	Not reported	Studied during oesophagectomy surgery Other treatment not reported	Intervention group 1 68 (±2) Intervention group 2 61 (±2) Control group 74 (±2)	1 (10) 2 (17) 1 (10)	Indirect calorimeter, E-COVX, GE Healthcare/Datex-Ohmeda
Sato et al. (1993) [53]	Japan	Before-after	19	Esophageal	TNM 0/1/2A/2B Transhiatal oesophagectomy 2/2/1/0 Transthoracic oesophagectomy 1/3/6/4	Pre-post oesophagectomy Other treatment not reported	Transhiatal oesophagectomy (*n =* 5) 72 (SEM 3) Transthoracic oesophagectomy (*n =* 14) 59 (SEM 3)	0 (0)	Indirect calorimeter (portable), RM300, Minato Medical Science
Sato et al. (1997) [54]	Japan	Before-after	35	Esophageal	Not reported	Pre-/post esophagectomy Other treatment not reported	62 (range 40–76)	0 (0)	Indirect calorimeter (portable), RM300, Minato Medical Science
Shinsyu et al. (2020) [41]	Japan	Cross-sectional	32	Esophageal (*n =* 17) Gastric (*n =* 15)	O: 6/1/6/4 G: 7/4/1/3	Mix of treatment naïve, or > 1 mo since chemo/radiotherapy	O: 65 (± 9) G: 65 (± 8)	1 (6) 4 (3)	Indirect calorimeter, Aeromonitor AE310S, Minato Medical Science
Sugihara et al. (2014) [83]	Japan	Before-after	18	Liver	Not reported	Pre-/post liver resection Other treatment not reported	67 (64, 73)^4^	3 (17)	Indirect calorimeter, AE-300S respiratory gas analyser, Minato Medical Science
Sukkar et al. (2003) [75]	Italy	Cross-sectional	13	Gastric	“Advanced stage” 0 patients with metastases	Awaiting surgical resection At least 30 d since last chemo/radiotherapy treatment	66 (±11)	0 (0)	Indirect calorimeter, Deltatrac, Datex
Tashiro et al. (1999) [70]	Japan	Before-after	3	Esophageal	Not reported	Pre-/post esophageal resection Other treatment not reported	52.7 (±3.8)	1 (33)	Indirect calorimeter, Metabolic Gas Monitor (MGM) II, Utah Medical
Terepka et al. (1956) [43]	USA	Noncontrolled trial	1	Pancreatic	Not reported	Not reported	60	0 (0)	Indirect calorimeter, standard Benedict-Roth apparatus
Thomson et al. (1990) [64]	South Africa	Cross-sectional	15^6^	Esophageal	0 patients with metastases	Awaiting surgical resection Other treatment not reported	Male (*n =* 9)^6^55 (±11) Female (*n =* 5) 6 54 (±9)	5 (33)^6^	Indirect calorimeter, Horizon Metabolic Measuring Cart, Beckman Instruments
Vaisman et al. (2012) [66]	Israel	Cross-sectional	45	Pancreatic	Both resectable and unresectable disease	Group 1 (*n =* 15) had prior surgery within 1-6 mo Group 2 (*n =* 30) had not had surgery Other treatment not reported	Not reported	Not reported	Indirect calorimeter, Deltatrac Metabolic Monitor, Datex
Wang et al. (2010) [55]	China	RCT	92	Gastric	Not reported	Post gastrectomy No prior chemo/radiotherapy	Intervention group (*n =* 45) 58.8 (±9.7) Control group (*n =* 47) 56.9 (±9.2)	13 (29) 18 (38)	Indirect calorimeter, MedGraphic
Wigmore et al. (1995) [61]	UK	Controlled trial	16	Pancreatic	Not reported	No surgery within 2 mo prior to study Other treatment not reported	60 (SEM 23)	6 (40)	Indirect calorimeter, Deltatrac, S&W Vickers
Wigmore et al. (1996) [56]	UK	Noncontrolled trial	18	Pancreatic	0/2/7/9	No prior chemo/radiotherapy *n =* 7 endoscopic stent insertion, *n =* 6 biliary bypass surgery (all > 4 wk before study)	Not reported	Not reported	Indirect calorimeter, Deltatrac, S&W Vickers
Wigmore et al. (1997) [84]	UK	Cross-sectional	35	Pancreatic	Not reported	*n =* 8 endoscopic stent insertion, *n =* 13 biliary bypass surgery (all > 4 wk before study) Other treatment not reported	High CRP group (*n =* 19) 65 (57, 69)^4^^,^^7^ Normal CRP group (*n =* 16) 66 (54, 72)^4^^,^^7^	Not reported	Indirect calorimeter, Deltatrac, S&W Vickers
Witvliet-van Nierop et al. (2017) [79]	Netherlands	Cross-sectional	16	Pancreatic	“Locally advanced”	*n =* 14 had prior treatment: combinations of chemotherapy (*n =* 7), percutaneous transhepatic cholangiography biliary drain or plastic endoprostheses (*n =* 5), and gastro- and/or hepaticojejunostomy (*n =* 6)	60.3 (± 9.5)	8 (50)	Indirect calorimeter, Vmax Encore n29, Viasys
Wu et al. (2013) [62]	China	Cross-sectional	56	Esophageal	Not reported/All stages	Newly diagnosed No prior chemo/radiotherapy, prior surgical intervention unclear	60.8 (±9.05)	0 (0)	Indirect calorimeter, CPX Ultima, MedGraphics
Wu et al. (2017) [88]	China	RCT	73	Esophageal	Intervention group 8/11/18/0 Control group 7/14/15/0	Immediately post esophagectomy *n =* 11 had prior “neoadjuvant treatment,” no further details reported	Intervention group (*n =* 37) 53.2 (±12.6) Control group (*n =* 36) 58.3 (±11.7)	11 (30) 12 (33)	Indirect calorimeter, QUARK RMR, Cosmed
Yatabe et al. (2014) [81]	Japan	Cross-sectional	15	Esophageal	2/2/8/3	During esophagectomy *n =* 13 (87%) had prior chemotherapy, no prior radiotherapy	66 (±10)	3 (20)	Indirect calorimeter, Engstrom device (ventilator and indirect calorimeter), GE Healthcare
Yoshikawa et al. (2001) [48]	Japan	Cross-sectional	17	Gastric	5/2/3/7	Not reported	Not reported	Not reported	Indirect calorimeter, Deltatrac Metabolic Monitor, Datex

B, biliary; BCLC, Barcelona Clinic Liver Cancer staging system [89]; EE, energy expenditure; ESD, endoscopic submucosal dissection; G, gastric; IOM, Institute of Medicine; IV, intravenous; mREE, measured resting energy expenditure; N/A, not applicable; O, esophageal; P, pancreatic; POD, postoperative day; pREE, predicted resting energy expenditure; RCT, randomized controlled trial.

1 participants with upper gastrointestinal cancers with reported endpoint data only;

2 data reported as median (range);

3 inconsistent sample size figures, reported as *n =* 16 with 10 men and 9 women;

4 data reported as median (interquartile range);

5 baseline data provided for separate treatment arms of RCT rather than whole cohort;

6 inconsistent sample size figures, reported as *n =* 15 with 9 men and 5 women;

7 high CRP group: ≥ 10mg/L, low CRP group: < 10mg/L.

A total of 2,125 participants with upper GI cancer were enrolled in the included studies (Table 1); 660 participants with esophageal cancer, 590 with gastric cancer, 416 with pancreatic cancer, 400 with liver cancer, and 17 with bile duct cancer. In 4 studies, the upper GI cancer types were grouped: 28 participants from 2 studies had esophageal or gastric cancer [36, 40], 9 participants in one study had “hepatobiliary” cancer [32], and 5 participants in one study had “pancreatic/biliary” cancer [39]. Sample sizes of upper GI cancer cohorts varied and ranged from one participant [42, 43] to 432 participants [6]; 29 studies (51%) involved 20 or fewer participants with upper GI cancer, 27 studies (47%) involved 21 to 100 participants, and one study (2%) involved more than 100 participants (432 participants).

Additional participant and study characteristics are presented in Supplementary Files 2 and 3. BMI was reported (or able to be calculated) for 31 studies (54%); group level BMI was within the healthy range (18.5–24.99kg/m^2^) or overweight/preobese range (25–29.99kg/m^2^) for all of these studies [91]. For 18 studies (32%), only weight data was available, and in 3 studies, data were unable to be separated by upper GI cancer type for BMI (*n =* 2 [35, 36]) or weight (*n =* 1 [32]). Five studies (9%) did not report participant BMI or weight [[44], [45], [46], [47], [48]].

There was heterogeneity between studies in relation to the clinical setting at the time of energy expenditure assessment. Baseline energy expenditure was measured before surgery and/or medical cancer treatment in 36 studies, during cancer resection surgery in one study, during the postoperative period in 2 studies, and in mixed cohorts of participants who were pre-, mid-, or postcancer treatment at the time of assessment in 6 studies. The clinical setting was not stated or was unclear in 11 studies (19%). In 30 studies (53%), only a baseline assessment of REE was conducted. Follow-up measures of energy expenditure were reported in 27 studies (47%) (*n =* 27 REE only, *n =* 1 REE and TEE), ranging from one day [37, 44, 46, [49], [50], [51], [52], [53], [54], [55]] to 3 mo [56] after the baseline assessment.

### Measurement of energy expenditure

REE data was expressed as kcal/day in 42 studies, kcal/kg/day in 32 studies, and kcal/kg FFM/day in 13 studies; 25 studies (44%) reported more than one of these measures. Moses et al. additionally measured TEE using DLW (expressed as kcal/day) in a cohort of 24 patients with pancreatic cancer [34]. Assessments of FFM were conducted using bioelectrical impedance analysis in 8 studies [6, 41, [57], [58], [59], [60], [61], [62]], triceps skinfold thickness in 2 studies [63, 64], total body water using isotope dilution in one study [65] and dual-energy x-ray absorptiometry in one study [66]. Table 2 [[6], [32], [34], [35], [36], [37], [38], [39], [40], [41], [42], [43], [44], [45], [46], [47], [48], [49], [50], [51], [52], [53], [54], [55], [56], [57], [58], [59], [60], [61], [62], [63], [64], [65], [66], [67], [68], [69], [70], [71], [72], [73], [74], [75], [76], [77], [78], [79], [80], [81], [82], [83], [84], [85], [86], [87], [88]] presents a summary of the number of studies reporting each of the outcomes of interest (kcal/day, kcal/kg/day, kcal/kg FFM/day) by cancer type.

**TABLE 2:** Summary of scoping review results: number of studies reporting key outcomes.

Cancer type	Oesophageal	Gastric	Pancreatic	Bile duct	Liver
Total studies, n (total participants)	25 (n = 660) Ref: [6,32,[36], [37], [38], [39], [40], [41],[47], [48], [49],^52^,[54], [55], [56],^64^,^66^,^70^,^72^,^79^,[93], [94], [95],^123^,^126^]	18 (n = 590) Ref: [6,32,[35], [36], [37], [38], [39], [40], [41],^46^,^50^,^51^,^53^,^57^,^67^,^69^,^71^,^77^]	19 (n = 416) Ref: [6,32,[34], [35], [36],^39^,^43^,^44^,[58], [59], [60], [61],^63^,^68^,^74^,^78^,^82^,^84^,^106^]	4 (n = 17) Ref: [32,35,39,70]	11 (n = 400) Ref: [32,35,39,62,65,73,75,76,96,124,125]
Studies reporting energy expenditure values (n)	**kcal/day**				
	17 Ref: [6,37,[39], [40], [41],^48^,^49^,^52^,^64^,^66^,^70^,^72^,[93], [94], [95],^123^,^126^]	18 Ref: [6,35,37,[39], [40], [41],^50^,^51^,^53^,^57^,^67^,^69^,^77^]	13 Ref: [6,34,35,39,43,44,59,60,63,68,74,82,84]	2 Ref: [35,39]	9 Ref: [35,39,62,65,73,75,76,124,125]
	**kcal/kg/day**				
	15 Ref: [[38], [39], [40], [41],^47^,^52^,[54], [55], [56],^64^,^66^,^70^,^72^,^79^,^94^]	10 Ref: [35,[38], [39], [40], [41],^51^,^53^,^67^,^71^,^77^]	11 Ref: [35,39,43,[58], [59], [60], [61],^63^,^74^,^78^,^106^]	2 Ref: [35,39]	4 Ref: [35,39,65,73]
	**kcal/kg FFM/day**				
	4 Ref: [6,41,64,66]	3 Ref: [6,41,67]	7 Ref: [6,[59], [60], [61],^63^,^68^,^82^]	0	2 Ref: [62,65]
Studies comparing EE in upper GI cancer with non-cancer controls (n)	6 Ref: [6,40,64,66,70,79]	4 Ref: [6,40,50,77]	7 Ref: [6,59,61,63,68,74,78]	0	5 Ref: [62,65,73,75,76]
Number of studies where REE was higher (>), similar to ( =), or lower (<) than non-cancer controls at baseline, n (total participants)	**kcal/day**				
	cancer > controls 1 (n = 56) Ref: [64]	–	cancer > controls 1 (n = 16) Ref: [63]	–	cancer > controls 3 (n = 82) Ref: [62,65,73]
	cancer = controls 4 (n = 187)^1^ Ref: [6,40,70], [66] females only	cancer = controls 4 (n = 208)^1^ Ref: [6,40,50,77]	cancer = controls 3 (n = 151) Ref: [6,59,74]	–	cancer = controls 2 (n = 113) Ref: [75,76]
	cancer < controls 1 (n = 9) Ref: [66] (males only)	–	cancer < controls 1 (n = 45) Ref: [68]	–	–
	**kcal/kg/day**				
	cancer > controls 2 (n = 64) Ref: [64,70]	cancer > controls 1 (n = 13) Ref: [77]	cancer > controls 4 (n = 60) Ref: [59,61,63,74]	–	cancer > controls 2 (n = 69) Ref: [65,73]
	cancer = controls 3 (n = 44)^1^ Ref: [40,66,79]	cancer = controls 1 (n = 24)^1^ Ref: [40]	cancer = controls 1 (n = 15) Ref: [78]		–
	**kcal/kg FFM/day**				
	cancer > controls 2 (n = 206) Ref: [6,64]	cancer > controls 1 (n = 154) Ref: [6]	cancer > controls 4 (n = 181) Ref: [6,59,61,63]	–	cancer > controls 1 (n = 12) Ref: [65]
	cancer = controls 1 (n = 15) Ref: [66]	–	cancer = controls 1 (n = 45) Ref: [68]	–	cancer = controls 1 (n = 13) Ref: [62]

Studies comparing measured^2^ EE with predicted EE using equations (n)	15 Ref: [6,32,[36], [37], [38], [39],^41^,^49^,^52^,^64^,^70^,^79^,[93], [94], [95]]	12 Ref: [6,32,[36], [37], [38], [39],^41^,^46^,^50^,^51^,^67^,^77^]	8 Ref: [6,32,34,36,39,43,68,84]	2 Ref: [32,39]	8 Ref: [32,39,62,65,73,75,76,96]
Number of studies where measured EE was higher (>), similar to ( =), or lower (<) than predicted EE, n (total participants)	**mREE > pREE** 1 (n = 30) Ref: [95]	–	**mREE > pREE** 1 (n = 24) Ref: [34]	–	**mREE > pREE** 2 (n = 41) Ref: [62] well-nourished group only, [75]
	**mREE = pREE** 3 (n = 35) Ref: [70,79,93] intervention groups only	**mREE = pREE** 1 (n = 22) Ref: [67]	–	–	**mREE = pREE**2 (n = 38) Ref: [62] malnourished group only, [75]
	**mREE < pREE** 2 (n = 25) Ref: [93] non-intervention group only, [94]	–	**mTEE < pTEE** 1 (n = 24) Ref: [34] (Schofieldx1.5 stress factor)	–	

Number of studies where participants were hyper-, normo-, or hypometabolic according to mREE/pREE ratio, n (total participants)	**Hypermetabolic (mREE/pREE >110%)**				
	7 (n = 153)^3^ Ref: [6,32,36,38,49,64,95]	5 (n = 127)^3^ Ref: [6,32,36,38,46]	5 (n = 101) Ref: [6,32,43,68,84]	1 (n = 1) Ref: [32]	2 (n = 13) Ref: [32,65]
	**Normometabolic (mREE/pREE 90–110%)**				
	9 (n = 207)^3^ Ref: [6,32,36,37,39,41,52,70,79,95]	8 (n = 190)^2^ Ref: [6,32,36,37,39,41,50,51]	5 (n = 96)^4^ Ref: [6,32,36,39,68]	2 (n = 6)^4^ Ref: [32,39]	5 (n = 193) Ref: [32,39,73,76,96]
	**Hypometabolic (mREE/pREE <90%)**				
	4 (n = 41) Ref: [6,32,94,95]	2 (n = 22) Ref: [6,32]	3 (n = 22) Ref: [6,32,36]	1 (n = 7) Ref: [32]	1 (n = 7) Ref: [32]

EE, energy expenditure; FFM, fat-free mass; mREE, measured resting energy expenditure; pREE, predicted resting energy expenditure; mTEE measured total energy expenditure; pTEE predicted total energy expenditure.

1 includes 24 participants with ‘oesophageal or gastric cancer’ [40], categorised under both cancer types.

2 studies where EE was measured using a reference method, i.e. indirect calorimetry or doubly labelled water.

3 includes two participants with ‘oesophageal or gastric’ cancer [36], categorised under both cancer types.

4 includes five participants with ‘pancreatic/biliary tract’ cancer [39], categorised under both cancer types.

Detailed energy expenditure data stratified by cancer type are presented in Supplementary File 2, showing a wide range of values for each of these outcomes within a variety of clinical and experimental conditions. For example, the ranges of reported baseline weight-adjusted REE (kcal/kg/day) were 20.2 to 27.3 for esophageal cancer, 20.2 to 31.2 for gastric cancer, 24.0 to 29.0 for pancreatic cancer, 23.7 to 30.7 for bile duct cancer, and 20.6 to 25.1 for liver cancer. Four studies of cohorts involving people with both upper GI and non-upper GI cancers investigated the difference in REE between cancer types [6, 35, 39, 41]; in 3 of these studies, there was no significant difference between cancer types in REE expressed as kcal/day [6, 35, 41], kcal/kg [35, 41], or kcal/kg FFM [41]. In the study by Omagari et al. [39], the difference in weight-adjusted REE between upper GI and nonupper GI cancers (esophageal, gastric, pancreatic/bile duct, liver or colorectal) appeared significant (*P*=0.001); the difference was attributed to a higher REE in patients with gastric cancer (24.8 kcal/kg/day, *n =* 20) compared with liver cancer (20.6 kcal/kg/day, *n =* 37). The difference in REE between upper GI cancer types alone was reported by Hioki et al. [37], where baseline REE in kcal/day was comparable between participants with esophageal (*n =* 10) and gastric cancers (*n =* 10) (*P*>0.05). In 12 studies, REE was measured before and after surgical intervention (only in studies of esophageal or gastric cancer); 7 of these studies reported a significant increase in REE postsurgery in at least one follow-up time point (total *n =* 226 participants) [[49], [50], [51], 53, 54, 67, 68], one study reported a significant decrease in REE in patients with gastric cancer receiving a placebo (*n =* 10) and a nonsignificant change in postsurgical REE for patients receiving daily ghrelin infusions (*n =* 10) [69], and the remaining 4 did not report on the significance of change in REE from before to after surgery [46, 52, 55, 70].

### Energy expenditure compared with noncancer controls

REE of people with upper GI cancers was compared with noncancer controls in 19 studies (33% of included studies), involving esophageal cancer in 6 studies (*n =* 268 participants including 24 “esophageal or gastric”), gastric cancer in 4 studies (*n =* 208 participants including 24 “esophageal or gastric”), pancreatic cancer in 7 studies (*n =* 248 participants), and liver cancer in 5 studies (*n =* 195 participants). An overview of these comparisons grouped by cancer type is presented in Table 2. Unadjusted REE was significantly higher than noncancer controls in 5 studies (*n =* 154 participants, range 113–363 kcal/d higher) [[60], [61], [62], [63], 71], significantly lower than noncancer controls in 2 studies (*n =* 54 participants, range 242–311 kcal/day lower) [66, 64], and not statistically different in 10 studies (*n =* 635 participants) [6, 40, 48, 57, 64, 68, [72], [73], [74], [75]]. When adjusted for body weight, REE was significantly higher than noncancer controls in 9 studies (*n =* 206 participants, range 1.13–6.5 kcal/kg/d higher) [57, 59, [61], [62], [63], 68, 71, 72, 75], and not statistically different in 4 studies (*n =* 59 participants) [40, 64, 76, 77]; these body weight-adjusted results differed from unadjusted REE in comparison with noncancer controls in 4 studies, moving from not statistically different (unadjusted) to significantly higher (weight-adjusted) in 3 studies (*n =* 31, esophageal, liver, pancreatic [57, 68, 72]), and from significantly lower (unadjusted) to not statistically different (weight-adjusted) for men in one study (*n =* 9, esophageal [64]).

REE of patients with cancer adjusted for FFM (kcal/kg FFM) was compared with noncancer controls in 9 studies (total *n =* 625) [6, 57, 58, [60], [61], [62], [63], [64], 66]; it was significantly higher than noncancer controls in 6 studies (*n =* 553 participants, range 1.26–27.9 kcal/kg FFM/d higher) [6, 59, 57, [63], [64], [65]], and not statistically different in 3 studies (*n =* 73 participants) [60, 64, 66]. Comparisons using FFM-adjusted REE differed from unadjusted REE in 5 studies (total *n =* 521, 83%) [6, 57, 60, 64, 66], moving from not statistically different (unadjusted) to significantly higher (FFM-adjusted) in 2 studies (*n =* 448, esophageal, gastric, pancreatic [6, 57]), from significantly lower (unadjusted) to not statistically different (FFM-adjusted) in 2 studies (*n =* 54, esophageal, pancreatic [64, 66]), and from significantly higher (unadjusted) to not significantly different in one study (*n =* 13, liver [60]). Further details of the study design, participant characteristics, and statistical comparisons between groups stratified by cancer type are presented in Supplementary File 2.

### Measured energy expenditure compared with predictive equations

Thirty-one studies (54% of included studies) compared energy expenditure measured using indirect calorimetry (*n =* 31 studies) and/or DLW (*n =* 1 study), with energy expenditure derived from at least one predictive equation. The Harris Benedict equation [92] was used in most of these studies (*n =* 29 studies, 94%), the Schofield equation [93] was used in 3 studies [34, 73, 78], the FAO/WHO/UNU equation [94] was used in 2 studies [73, 79], the Mifflin-St Jeor equation [95] was used in 2 studies [73, 78], and the Cunningham equation [96] in 2 studies [73, 78]. Bauer et al. [78] additionally compared measured REE with the Owen [97, 98] and Wang equations [99] and the 20kcal/kg ratio, whereas Henz et al. [73] additionally compared measured REE with the WHO 2000 [100], Institute of Medicine [101], and McArdle equations [102]. Table 2 outlines a summary of these comparisons by cancer type.

The statistical difference between measured and predicted energy expenditure was reported in 9 studies [6, 34, 60, 65, 68, 73, 77, 80, 81]. Measured energy expenditure was significantly higher than predicted in 4 studies (*n =* 95 participants, range 111–261 kcal/d higher) [34, 60, 73, 82], significantly lower than predicted in 3 studies (*n =* 49 participants, range 130–206 kcal/day lower) [34, 80, 81], and not statistically different in 6 studies (*n =* 95 participants) [60, 65, 68, 73, 77, 80]; there was some overlap, as 4 of these studies found different results for either different sub-groups of participants [60, 68], energy expenditure assessment method [34], or different predictive equations [73].

Measured REE expressed as a percentage of predicted (mREE/pREE, %) was reported in 23 studies, either for the study cohort or the number of participants in each metabolic group. Participants were found to be “hypermetabolic” in 12 studies (*n =* 403 participants) [6, 32, 36, 38, 42, 44, 47, 62, 63, 66, 79, 82], “normometabolic” in 16 studies (*n =* 667 participants) [6, 32, 36, 37, 39, 41, [48], [49], [50], 66, 68, 71, 74, 77, 82, 83], and “hypometabolic” in 5 studies (*n =* 99 participants) [6, 32, 36, 81, 82].

In the only study to measure TEE using DLW, Moses et al. reported that measured TEE in patients with pancreatic cancer was significantly lower than predicted TEE using the Schofield equation with an activity factor of 1.5 [34]. Only one other study reported predicted TEE, applying an activity factor of 1.3 to the FAO/WHO/UNU equation [79]; the predicted TEE was reported as 590 kcal (IQR 276–736) higher than “measured TEE” (i.e., measured REE which had also been multiplied by 1.3). Further details of the study design, participant characteristics, and the comparisons of measured and predicted energy expenditure stratified by cancer type are provided in Supplementary File 3.

### Subgroup analyses

Two studies conducted further subgroup analyses examining differences in energy expenditure between groups of patients with upper GI cancer with various sub-characteristics [50, 62]. In a study of 75 people with esophageal cancer, Kudo et al. [50] reported that a higher total resection area (≥6.9 cm^2^) during endoscopic submucosal dissection was associated with a significant increase in REE; this study found no differences in REE between participants stratified by age (above vs. below 65 y), BMI (above vs. below 25 kg/m^2^), presence/absence of sarcopenia defined using sex-specific psoas muscle index cut points, or duration of procedure (less than vs. greater than 75 min). Wu et al. [62] found that measured REE (kcal/day, kcal/kg/day, and kcal/kgFFM/day) was significantly higher in weight-losing patients with esophageal cancer (*n =* 24) compared with those who were weight stable (*n =* 32), with no between-group differences noted in age, BMI, or FFM.

## Discussion

The purpose of this scoping review was to summarize the evidence base with regard to objectively measured TEE and REE in people with upper GI cancers and the comparison of these measurements with noncancer controls and/or predicted energy expenditure derived from equations. To our knowledge, this is the first study to systematically synthesize the findings of studies reporting on energy expenditure in upper GI cancer, reviewing a large and diverse body of literature spanning several decades.

All 57 included studies measured REE using indirect calorimetry, and one study additionally measured TEE using DLW. The most studied upper GI cancer type was esophageal (25 studies), followed by pancreatic cancer (19 studies), whereas only 4 studies investigated energy expenditure in bile duct cancer. There was substantial between-study variation in daily energy expenditure (both unadjusted and adjusted for body weight or FFM), both within and between cancer types. Evidence regarding measured compared with predicted energy expenditure and differences in measured energy expenditure between those with and without cancer are currently inconsistent.

Understanding energy requirements for people with cancer is crucial for the development of effective nutrition interventions. The notion that energy expenditure is elevated in people with cancer stems from the evidence of comparisons with noncancer cohorts, where significant elevations in REE have been observed in various cancer types such as pancreatic, gastric, esophageal, and lung cancers [6, 103, 104]. In the present review, there was heterogeneity in the REE of people with upper GI cancer compared with noncancer controls in the 19 studies where this comparison was reported; REE was significantly higher than noncancer controls in 11 studies (total *n =* 651), significantly lower than noncancer controls in 2 studies (total *n =* 54) [64, 66], and 14 studies reported no difference (total *n =* 723). These findings highlight that the direction of REE alterations in upper GI cancers is not uniform [32], which has implications for nutrition assessment and intervention for this population and underscores the importance of individualized nutrition appraisal and regular monitoring of intervention effects. Notably, there were within-study differences in the comparisons of REE in people with and without cancer in 7 studies, depending on the unit of REE expression [6, 57, 60, 64, 66, 68, 72]. These changes in the significance of REE differences between groups after adjusting for FFM and/or body weight demonstrate the importance of consideration of participant phenotypes and body composition when examining the effect of cancer on REE. As FFM is a major determinant of REE [6, 14, 62, 105], the presence of low skeletal muscle mass associated with aging, inactivity, and/or the impact of cancer treatment might be expected to result in reduced REE [14, 106]. Conversely, as absolute REE increases with higher body weight due to an increase in FFM [106], there is potential for the inclusion of participants with high BMI to elevate the unadjusted REE of the cohort with cancer or the noncancer controls [14]. This may explain some of the variations in results, but it is difficult to verify with the available data in this scoping review. BMI data was available for 31 studies, with 3 of these studies reporting measures of variance indicating that some participants may have been obese (BMI ± 30kg/m^2^) (Supplementary File 2) [39, 50, 73]; it is possible that this may also be the case for some of the 26 remaining studies where BMI was not reported. For instance, in the study by Henz et al. (mean BMI 27.0 ±4.0) [73], the unadjusted REE of people with liver cancer was compared with that of noncancer controls with no significant difference reported; further analysis of this data would be required to determine whether a comparison of weight or FFM-adjusted REE would change this result. An important consideration outlined in prior literature reviews is the variation in proportional increase of different types of FFM with increasing weight (i.e., greater increase in bone and skeletal muscle than metabolically active organs such as the heart and brain), meaning that the often-utilized ratio approach to adjustment for FFM (REE/kg FFM) or body weight (REE/kg body weight) is theoretically flawed [14, 106]. Regression modeling controlling for confounding variables (e.g., age, sex, fat mass) has been proposed as a more appropriate method of comparing REE between groups [14, 106]. In future studies conducting similar analyses, participant BMI data, as well as measures of REE that have been adjusted using appropriate modeling, are needed to strengthen the interpretation of the observed impact of cancer on REE.

Measures of REE using indirect calorimetry comprise most, but not all, of an individual’s TEE; physical activity also contributes (20–30%). Only one study in this review measured TEE in upper GI cancer using DLW (34). In this study by Moses et al., measured TEE was significantly lower than predicted using the Schofield equation with an activity factor of 1.5. The authors proposed this was related to reduced physical activity in their cohort of 24 patients with pancreatic cancer; the mean physical activity level was reported as 1.24, lower than that of other healthy sedentary adults [107] and closer to that of individuals with cerebral palsy [108]. In a more recent study using DLW to assess TEE, the mean physical activity level was higher at 1.43 in 21 patients with colorectal cancer [109]. The principal clinical guidelines for nutrition interventions in cancer are broad and indicate that TEE for people with cancer can be assumed as similar to that of the noncancer population (25–30 kcal/kg/d) [110]). The level of evidence for this recommendation is “low” [110], and is made in acknowledgment of the heterogeneity in reported assessments of energy expenditure in the evidence base to date, attributed to the wide range of subconditions in this population (e.g., cancer type and stage of disease [14]). In clinical practice, the application of even a low activity factor of 1.2 times the REE [111] would exceed the top end of this recommended range for any individuals with an REE of over 25 kcal/kg/d; data from this scoping review showed that baseline REE was greater than 25 kcal/kg/d in 11 studies involving patients with esophageal [38, 40, 52, 64, 70], gastric [35, 40, 75], bile duct [35], and pancreatic cancers [35, 59, 61, 84], suggesting that this recommendation could underestimate energy needs in some patients. There is a dearth of evidence regarding TEE assessed using DLW in cancer, and further research is needed to understand the impact of cancer location and physical activity on TEE [109].

The measured REE of different upper GI cancer types was heterogenous, both within and between studies. Differences in energy expenditure between studies investigating the same cancer types may be attributed to population characteristics, clinical conditions, or measurement protocol. The hypothesis for cancer type affecting energy expenditure has been discussed in the literature and relates to the metabolic demand of cancer-affected organs potentially influencing metabolism [14, 104]. Five studies included in this review examined the differences in REE between cancer types. In one study [39], the difference in energy expenditure between upper GI cancer types was statistically significant (gastric > liver), but in most studies where this comparison was made, the difference between cancer types was nonsignificant [6, 35, 37, 41]. A recent scoping review of energy expenditure in gynecological cancers yielded only 5 studies (all with sample sizes <100, total *n =* 253) but also demonstrated heterogeneity in measured energy expenditure [112]; one study in that review compared energy expenditure between cancer types, reporting a significant difference in energy expenditure between participants with ovarian and cervical cancers [113]. Due to the heterogeneity in the evidence base of study methodologies and outcomes, the effect of cancer type on energy expenditure remains uncertain [104, 114].

In the clinical setting, the use of predictive equations to estimate the energy requirements of patients with cancer is common practice. Thirty-one studies in this review compared measured REE and/or TEE against predictive equations, with most of these studies using the equation developed by Harris and Benedict et al. [92]. There was significant heterogeneity in the accuracy of predictive equations reported; measured energy expenditure was found to be lower, similar, or higher than predicted across all cancer types. The study by Pagano et al. reported similar findings in studies of gynecological cancers [112]. Difficulties in the determination of energy needs in upper GI cancer pose a challenge for researchers or clinicians seeking to provide effective interventions for this nutritionally vulnerable group. The limitations of predictive equations for accurate estimation of energy requirements in cancer have been well documented, with both over- and underestimation of energy requirements observed [15, 22, 35, 78, 112]. Common predictive equations were derived from studies of healthy cohorts [92], limiting their applicability to individuals with diseases such as cancer. Nonspecific recommendations and predictive equations usually incorporate basic demographic elements that affect energy expenditure, such as age [115] and sex [116] (e.g., Harris Benedict and Schofield equations [80, 81]), and body weight (e.g., ESPEN 25–30kcal/kg [114]). Improvements to predictive equations which account for FFM may assist in improving the accuracy of energy expenditure estimation. Ozorio et al. [116] have recently proposed a new predictive equation derived from energy expenditure data of 109 people with advanced GI cancer, which incorporates sex and FFM measured through bioelectrical impedance rather than weight or age; however, this formula is yet to be validated.

There was substantial variation in clinical demographics, experimental or observational conditions, and the design of the studies included in this scoping review. The degree of detail and inclusion of reporting of important clinical factors also varied, with one-quarter of studies not reporting cancer stage and one-fifth of studies not reporting treatment status of participants. Our findings align with the scoping review by Pagano et al.*,* who observed that one study (20%) did not report on cancer stage, and 2 studies (40%) did not report on cancer treatment at the time of energy expenditure measurements. Description of cancer stage is important, as higher tumor burden or advanced cancer stage is hypothesized to contribute to elevations in REE through increased glucose consumption [6, 117], although evidence to the contrary has also been reported [14, 67, 118]. The influence of surgical interventions on REE is an additional confounder in this study, as the occurrence and/or timing of surgical intervention prior to energy expenditure assessment was heterogenous and, in some studies, not described [6, 32, 39, 43, 48, 60, 62, 63, 73, 74]. Evidence suggests metabolic stress associated with surgical intervention is variable and can increase REE by up to 10% or more in the early postoperative period [[119], [120], [121]]. Of the studies in this review comparing pre- and postoperative REE, most reported a significant increase in REE [[49], [50], [51], 53, 54, 67, 68], highlighting that postoperative treatment status should be considered when determining energy needs. In a recent systematic review of the effect of chemotherapy on REE in patients with cancer [122], there was some indication that this treatment may reduce REE (potentially related to suppression of tumor activity and/or reduction in FFM); however, no conclusions could be drawn due to the small and heterogenous evidence base [122]. In the present review, the influence of chemotherapy was difficult to elucidate from the varied descriptions of this factor within the included studies; therefore, the effect of chemotherapy on REE remains unclear. The breadth of variation in clinical and demographic characteristics of participants and years since publication within the included studies of this review precludes the formation of conclusions, particularly as advances in both cancer treatments and energy expenditure assessment techniques may affect measures of metabolism [104]. In future research, studies should provide clear descriptions of cancer stage and treatments to enhance understanding of energy expenditure in specific contexts and to inform the development of nutrition interventions.

Given the inconsistency of current results, we cannot confidently rely on any particular equation for the estimation of REE without further validation and research. The use of indirect calorimetry to accurately determine energy requirements in research and clinical practice has been recommended to enhance accuracy [15, 35, 123]. With advances in technology, indirect calorimeters are becoming increasingly affordable, and the development of portable devices also enhances their practicality [15, 124]. Studies assessing the validity of portable indirect calorimeters in cancer have reported suboptimal accuracy compared with traditional or whole-body calorimetry [125, 126], although repeated measurements were found to be reliable, meaning that changes in energy expenditure could be detected in these devices [125].

The broad search strategy utilized in this scoping review was a strength of this study; original research studies published at any time in any language were eligible for inclusion, ensuring that the maximum number of relevant studies were captured. The protocol for this review was prospectively registered, and the study has been reported according to the PRISMA-ScR framework. The outcomes of interest have been synthesized comprehensively, providing a critical summary of the existing evidence for energy expenditure in upper GI cancers and highlighting knowledge gaps. The volume and heterogeneity of reported data, as well as imprecision related to the small sample sizes of many included studies, limits the formation of definitive conclusions regarding energy expenditure in upper GI cancer. An additional limitation of this review is that potentially important data from additional studies where energy expenditure was not reported by cancer type (involving individuals with both upper GI and other cancer types) were excluded from this review (*n =* 17). Due to the scoping nature of this review, the studies included in this review were not subjected to formal quality and risk of bias assessment; however, variability in the rigor of the methodology and reporting between studies dating back as far as 1956 was evident.

## Conclusion

A systematic synthesis of available evidence regarding objective measures of energy expenditure in people with upper GI cancers has been presented in this scoping review. All included studies measured REE using indirect calorimetry, with substantial variation in energy expenditure values between cancer types and between studies. The measured REE of patients with upper GI cancer also varied in how it related to that of noncancer control groups and/or predicted energy expenditure using equations. Meta-analysis of participant-level data within cancer type and treatment/setting groups was outside the scope of this review, but future studies may reduce heterogeneity and enhance understanding of the effect of these factors on energy expenditure. Further research is also needed to understand TEE in upper GI cancer [13], which was only reported in one study using DLW [34]. Considering the known inaccuracies of predictive equations for estimating energy needs and the numerous variables that could potentially impact energy expenditure throughout the treatment trajectory, the incorporation of indirect calorimetry into upper GI cancer research and clinical practice is recommended. Future research studies should examine energy expenditure in homogenous cohorts to better understand the impact of cancer type, stage, body composition, and cancer treatment on energy requirements in upper GI cancer to guide the development of interventions in situations where indirect calorimetry is not feasible.

## Acknowledgments

LH, JP, JB, and KN designed research. LH, KN, JP, and JB conducted research. LH drafted the manuscript; all authors reviewed and revised the manuscript. All authors had primary responsibility for final content. All authors read and approved the final manuscript.

### Conflict of interest

All authors declare no conflicts of interest.

### Funding

The authors reported no funding received for this study.

## Appendix A Supplementary data

Multimedia component1 Multimedia component1

Multimedia component2 Multimedia component2

Multimedia component3 Multimedia component3
