# Supplementary material for: Energy Expenditure in Upper Gastrointestinal Cancers: a Scoping Review
Source: Adv Nutr. 2023 Aug 8;14(6):1307–25. doi: 10.1016/j.advnut.2023.08.002 (PMC10721480; doi:10.1016/j.advnut.2023.08.002)
Supplement: Multimedia component1 [file mmc1.docx]

**Database Search Strategies**

**Ovid MEDLINE**

#1 (upper gastrointestinal adj3 (cancer* or tumo?r* or neoplasm* or carcinoma* or malignan* or adenocarcin*)).mp

#2 (oesophag* adj3 (cancer* or tumo?r* or neoplasm* or carcinoma* or malignan* or adenocarcin*)).mp

#3 (esophag* adj3 (cancer* or tumo?r* or neoplasm* or carcinoma* or malignan* or adenocarcin*)).mp

#4 (stomach adj3 (cancer* or tumo?r* or neoplasm* or carcinoma* or malignan* or adenocarcin*)).mp

#5 (gastric adj3 (cancer* or tumo?r* or neoplasm* or carcinoma* or malignan* or adenocarcin*)).mp

#6 (pancrea* adj3 (cancer* or tumo?r* or neoplasm* or carcinoma* or malignan* or adenocarcin*)).mp

#7 (bil* adj3 (cancer* or tumo?r* or neoplasm* or carcinoma* or malignan* or adenocarcin*)).mp

#8 cholangiocarcinoma*.mp.

#9 (liver adj3 (cancer* or tumo?r* or neoplasm* or carcinoma* or malignan* or adenocarcin*)).mp

#10 (hepato* adj3 (cancer* or tumo?r* or neoplasm* or carcinoma* or malignan* or adenocarcin*)).mp

#11 (duoden* adj3 (cancer* or tumo?r* or neoplasm* or carcinoma* or malignan* or adenocarcin*)).mp

#12 (ile* adj3 (cancer* or tumo?r* or neoplasm* or carcinoma* or malignan* or adenocarcin*)).mp

#13 (jejun* adj3 (cancer* or tumo?r* or neoplasm* or carcinoma* or malignan* or adenocarcin*)).mp

#14 exp Esophageal Neoplasms/

#15 exp Stomach Neoplasms/

#16 exp Pancreatic Neoplasms/

#17 exp Bile Duct Neoplasms/

#18 exp Liver Neoplasms/

#19 exp Duodenal Neoplasms/

#20 exp Ileal Neoplasms/

#21 exp Jejunal Neoplasms/

#22 1 or 2 or 3 or 4 or 5 or 6 or 7 or 8 or 9 or 10 or 11 or 12 or 13 or 14 or 15 or 16 or 17 or 18 or 19 or 20 or 21

#23 energy expend*.mp.

#24 energy metabolism.mp.

#25 estimat* energy.mp.

#26 caloric expend*.mp.

#27 resting metabolic rate.mp.

#28 basal metabolic rate.mp.

#29 indirect calorimet*.mp.

#30 respiration calorimet*.mp.

#31 doubly label?ed water.mp.

#32 DLW.mp.

#33 TEE.mp.

#34 DEE.mp.

#35 TDEE.mp.

#36 (24h adj2 EE).mp.

#37 (24h adj2 energy expend*).mp.

#38 (total adj2 EE).mp.

#39 exp Basal Metabolism/

#40 exp Calorimetry, Indirect/

#41 23 or 24 or 25 or 26 or 27 or 28 or 29 or 30 or 31 or 32 or 33 or 34 or 35 or 36 or 37 or 38 or 39 or 40

#42 22 and 41

**Embase via Ovid**

#1 (upper gastrointestinal adj3 (cancer* or tumo?r* or neoplasm* or carcinoma* or malignan* or adenocarcin*)).mp

#2 (oesophag* adj3 (cancer* or tumo?r* or neoplasm* or carcinoma* or malignan* or adenocarcin*)).mp

#3 (esophag* adj3 (cancer* or tumo?r* or neoplasm* or carcinoma* or malignan* or adenocarcin*)).mp

#4 (stomach adj3 (cancer* or tumo?r* or neoplasm* or carcinoma* or malignan* or adenocarcin*)).mp

#5 (gastric adj3 (cancer* or tumo?r* or neoplasm* or carcinoma* or malignan* or adenocarcin*)).mp

#6 (pancrea* adj3 (cancer* or tumo?r* or neoplasm* or carcinoma* or malignan* or adenocarcin*)).mp

#7 (bil* adj3 (cancer* or tumo?r* or neoplasm* or carcinoma* or malignan* or adenocarcin*)).mp

#8 cholangiocarcinoma*.mp.

#9 (liver adj3 (cancer* or tumo?r* or neoplasm* or carcinoma* or malignan* or adenocarcin*)).mp

#10 (hepato* adj3 (cancer* or tumo?r* or neoplasm* or carcinoma* or malignan* or adenocarcin*)).mp

#11 (duoden* adj3 (cancer* or tumo?r* or neoplasm* or carcinoma* or malignan* or adenocarcin*)).mp

#12 (ile* adj3 (cancer* or tumo?r* or neoplasm* or carcinoma* or malignan* or adenocarcin*)).mp

#13 (jejun* adj3 (cancer* or tumo?r* or neoplasm* or carcinoma* or malignan* or adenocarcin*)).mp

#14 exp esophagus cancer/

#15 exp stomach cancer/

#16 exp pancreas cancer/

#17 exp hepatobiliary system carcinoma/

#18 exp hepatobiliary system cancer/

#19 exp duodenum cancer/

#20 exp ileum cancer/

#21 exp jejunum cancer/

#22 1 or 2 or 3 or 4 or 5 or 6 or 7 or 8 or 9 or 10 or 11 or 12 or 13 or 14 or 15 or 16 or 17 or 18 or 19 or 20 or 21

#23 energy expend*.mp.

#24 energy metabolism.mp.

#25 estimat* energy.mp.

#26 caloric expend*.mp.

#27 resting metabolic rate.mp.

#28 basal metabolic rate.mp.

#29 indirect calorimet*.mp.

#30 respiration calorimet*.mp.

#31 doubly label?ed water.mp.

#32 DLW.mp.

#33 TEE.mp.

#34 DEE.mp.

#35 TDEE.mp.

#36 (24h adj2 EE).mp.

#37 (24h adj2 energy expend*).mp.

#38 (total adj2 EE).mp.

#39 exp energy expenditure/

#40 exp indirect calorimetry/

#41 23 or 24 or 25 or 26 or 27 or 28 or 29 or 30 or 31 or 32 or 33 or 34 or 35 or 36 or 37 or 38 or 39 or 40

#42 22 and 41

**CINAHL plus**

S1 TI (“upper gastrointestinal” N3 (cancer* or tumo#r* or neoplasm* or carcinoma* or malignan* or adenocarcin*) or AB (“upper gastrointestinal” N3 (cancer* or tumo#r* or neoplasm* or carcinoma* or malignan* or adenocarcin*)

S2 TI (oesophag* N3 (cancer* or tumo#r* or neoplasm* or carcinoma* or malignan* OR adenocarcin*) or AB (oesophag* N3 (cancer* or tumo#r* or neoplasm* or carcinoma* or malignan* or adenocarcin*)

S3 TI (esophag* N3 (cancer* or tumo#r* or neoplasm* or carcinoma* or malignan* or adenocarcin*) or AB (esophag* N3 (cancer* or tumo#r* or neoplasm* or carcinoma* or malignan* or adenocarcin*)

S4 TI (stomach N3 (cancer* or tumo#r* or neoplasm* or carcinoma* or malignan* or adenocarcin*) or AB (stomach N3 (cancer* or tumo#r* or neoplasm* or carcinoma* or malignan* or adenocarcin*)

S5 TI (gastric N3 (cancer* or tumo#r* or neoplasm* or carcinoma* or malignan* or adenocarcin*) or AB (stomach N3 (gastric* or tumo#r* or neoplasm* or carcinoma* or malignan* or adenocarcin*)

S6 TI (pancrea* N3 (cancer* or tumo#r* or neoplasm* or carcinoma* or malignan* or adenocarcin*) or AB (pancrea* N3 (cancer* or tumo#r* or neoplasm* or carcinoma* or malignan* or adenocarcin*)

S7 TI (bil* N3 (cancer* or tumo#r* or neoplasm* or carcinoma* or malignan* or adenocarcin*) or AB (bil* N3 (cancer* or tumo#r* or neoplasm* or carcinoma* or malignan* or adenocarcin*)

S8 TI cholangiocarcinoma* or AB cholangiocarcinoma*

S9 TI (liver N3 (cancer* or tumo#r* or neoplasm* or carcinoma* or malignan* or adenocarcin*) or AB (liver N3 (cancer* or tumo#r* or neoplasm* or carcinoma* or malignan* or adenocarcin*)

S10 TI (hepato* N3 (cancer* or tumo#r* or neoplasm* or carcinoma* or malignan* or adenocarcin*) or AB (hepato* N3 (cancer* or tumo#r* or neoplasm* or carcinoma* or malignan* or adenocarcin*)

S11 TI (duoden* N3 (cancer* or tumo#r* or neoplasm* or carcinoma* or malignan* or adenocarcin*) or AB (duoden* N3 (cancer* or tumo#r* or neoplasm* or carcinoma* or malignan* or adenocarcin*)

S12 TI (ile* N3 (cancer* or tumo#r* or neoplasm* or carcinoma* or malignan* or adenocarcin*) or AB (ile* N3 (cancer* or tumo#r* or neoplasm* or carcinoma* or malignan* or adenocarcin*)

S13 TI (jejun* N3 (cancer* or tumo#r* or neoplasm* or carcinoma* or malignan* or adenocarcin*) or AB (jejun* N3 (cancer* or tumo#r* or neoplasm* or carcinoma* or malignan* or adenocarcin*)

S14 (MH “Esophageal Neoplasms+”)

S15 (MH “Stomach Neoplasms”)

S16 (MH “Liver Neoplasms+”)

S17 (MH “Biliary Tract Neoplasms+”)

S18 (MH “Pancreatic Neoplasms+”)

S19 (MH “Duodenal Neoplasms”)

S20 (MH “Ileal Neoplasms”)

S21 (MH “Jejunal Neoplasms”)

S22 S1 or S2 or S3 or S4 or S5 or S6 or S7 or S8 or S9 or S10 or S11 or S12 or S13 or S14 or S15 or S16 or S17 or S18 or S19 or S20 or S21

S23 TI “energy expend*” or AB “energy expend*”

S24 TI “energy metabolism” or AB “energy metabolism”

S25 TI “estimate* energy” or AB “estimate* energy”

S26 TI “caloric expend*” or AB “caloric expend*”

S27 TI “resting metabolic rate” or AB “resting metabolic rate”

S28 TI “basal metabolic rate” or AB “basal metabolic rate”

S29 TI “indirect calorimet*” or AB “indirect calorimet*”

S30 TI “respiration calorimet*” or AB “respiration calorimet*”

S31 TI “doubly label#ed water” or AB “doubly label#ed water”

S32 TI DLW or AB DLW

S33 TI TEE or AB TEE

S34 TI DEE or AB DEE

S35 TI TDEE or AB TDEE

S36 TI 24h N2 EE or AB 24h N2 EE

S37 TI total N2 EE or AB total N2 EE

S38 (MH “Basal Metabolism+”)

S39 (MH “Calorimetry”)

S40 S23 or S24 or S25 or S26 or S27 or S28 or S29 or S30 or S31 or S32 or S33 or S34 or S35 or S36 or S37 or S38 or S39

S41 S22 and S40
