# Supplementary material for: Energy Expenditure in Upper Gastrointestinal Cancers: a Scoping Review
Source: Adv Nutr. 2023 Aug 8;14(6):1307–25. doi: 10.1016/j.advnut.2023.08.002 (PMC10721480; doi:10.1016/j.advnut.2023.08.002)
Supplement: Multimedia component2 [file mmc2.docx]

**Supplementary File 2** Measured energy expenditure of people with upper gastrointestinal cancer, with comparison to non-cancer population (if reported)

| **Author, year** | **BMI (kg/m^2^)** | **Timing of EE assessment** | **Daily resting energy expenditure** | | | **Comparator cohort** | **Daily resting energy expenditure** | | |
| --- | --- | --- | --- | --- | --- | --- | --- | --- | --- |
|  | (mean ±SD) |  | kcal | kcal/kg | kcal/kg FFM | Sample size (n), demographics | kcal | kcal/kg | kcal/kg FFM |
| OESOPHAGEAL CANCER: COMPARISON TO NON-CANCER CONTROL | | | | | | | | | |
| Cao *et al*. (2010) | 22.53 (±3.0) | Once, prior to starting treatment | 1480 (±147)***^ns^*** | - | 32.38 (±4.56)^1^  **Higher than non-cancer control (p<0.05)** | Non-cancer control n = 642  Age and gender matched  Weight and BMI higher than cancer group (p<0.05) | 1448 (±188) | - | 30.31 (±4.07) |
| Klein *et al*. (1990) | Weight (kg)  58 (SEM 4) | Once, prior to starting treatment | - | 21.9 (SEM 1.3)*^ns^* | - | Control group 1, n=5  Cachectic patients (non-cancer)  Matched for gender and weight history | - | 26.3 (SEM 0.9) | - |
|  |  |  |  |  |  | Control group 2, n=10  Healthy volunteers, weight stable |  | 22.4 (SEM (0.6) | - |
| Okamoto *et al*. (2001) | 19.3 (±1.9) | Baseline (pre-surgery) | 1257 (±110)*^ns^* | 23.3 (±2.1)  **Higher than non-cancer control (p<0.05)** | - | Non-cancer control n=8  Age and gender matched  Weight and BMI higher than cancer group (both p<0.001) | 1438 (±204) | 20.4 (±1.6) | - |
|  |  | POD 7 |  | 27.3 (±3.5)  Higher than baseline (p<0.05) | - | - | - | - | - |
|  |  | POD 14 |  | Data in figure only  Not significantly different to baseline (p>0.05) | - | - | - | - | - |
| Rabito *et al*. (2013)^2^ | 20 (±3) | Once, pre-surgery | 1378 (±227)*^ns^* | 26 (±3) *^ns^* | - | Non-cancer control n=12  Majority female (66%)  Younger than cancer group (p<0.05)  Weight and BMI higher than cancer group (both p<0.05) | 1588 (±210) | 26 (±3) | - |
| Thomson *et al*. (1990) | Weight (kg)  M: 49 (±8)  F: 53 (±12) | Once, pre-surgery | M: 1448 (±134)  **Lower than male non-cancer controls (p=0.002)**  F: 1520 (±268)*^ns^* | M 28.7 (±3.6)*^ns^*  F: 29.4 (±2.9)*^ns^* | M: 33.7 (±4.8)^3,^ *^ns^*  F: 33.0 (±2.4)^3,^ *^ns^* | Non-cancer controls, n=17  Age, gender and height matched  Weight higher than cancer group (p-value not reported) | M: 1690 (±153)  F: 2063 (±277) | M: 28.4 (±2.6)  F: 30.6 (±4.8) | M: 33.9 (±2.4)  F: 32.0 (±4.5) |
| Wu *et al*. (2013) | 21.6 (±2.61) | Once, prior to starting treatment | 1595 (±325)  **Higher than non-cancer controls (p=0.037)** | 26.8 (±5.12)  **Higher than non-cancer controls (p<0.001)** | 57.3 (5.11)^1^  **Higher non-cancer controls (p<0.004)** | Non-cancer controls, n=30  Age and gender matched  Weight and BMI higher than cancer group (p=0.006, and p=0.002, respectively) | 1448 (±266) | 22.5 (±5.02) | 29.4 (±5.92) |
| **OESOPHAGEAL CANCER: BETWEEN-GROUP COMPARISONS** | | | | | | | | | |
| Sane *et al*. (2000) | Intervention group (n=9)  Post-surgical TPN without lipid, 6 days  Weight (kg)  53.9 (± 6.5) | Baseline (pre-surgery) | - | Results in Figure 1a |  | Oesophageal cancer in control arm of RCT, n=9  Age, gender and weight matched to intervention group  Post-surgical TPN containing lipid, 6 days | Results in Figure 1b | - | - |
|  |  | Daily on POD 1-6 |  | Intervention group: approximately 30-35 kcal/day from baseline to POD 6 |  |  | Control group: approximately 30-35 kcal/day from baseline to POD 6 |  |  |
| Satoh *et al*. (2018) | Intervention group 1 (n=10)  IV glucose 3g/hr with AA 1.2g/hr during surgery  22 (±3)  Intervention group 2 (n=12)  IV glucose 4.5g/hr with AA 1.8g/hr during surgery  20 (±3) | Once, during surgery | Intervention group 1  1230 (±228)*^ns^*  Intervention group 2  1317 (±282)  Higher than control group (p<0.05) | - | - | Oesophageal cancer in control arm of trial, n=10  IV therapy without glucose or AA during surgery  Gender matched, older than  Intervention Group 2 (p<0.05)  Similar weight and BMI to both intervention groups | 1012 (±153) | - | - |
| Sato *et al*. (1993) | Transhiatal oesophagectomy (THO)  Weight (kg)  56 (SEM 6)  Transthoracic oesophagectomy (TTO)  Weight (kg)  57 (SEM 2) | Baseline (pre-surgery  POD 1, 3, 5, 7 | - | Results in Figure 1  THO group:  Approx. 21.5kcal/kg at baseline, increase to approx. 26.3kcal/kg at POD 1-5, decrease to approx. 23.4kcal/kg at POD 7 (all changes non-significant, p>0.05)  TTO group:  Approx. 22.7kcal/kg at baseline, significant increase to approx. 29.9kcal/kg from POD 1-7 (p<0.05) | - | Between group comparison:  REE (kcal/kg/day) higher in TTO group than THO group at POD 7 (p<0.05), no significant difference at other time points | - | - | - |
| Wu *et al*. (2017) | Intervention group (n=37)  Supplemental PN in addition to EN post-surgery  23.0 (±3.2) | Daily on POD 1-4  Average of daily measures reported | 1710 (±468)*^ns^* | - | - | Oesophageal cancer in control arm of trial, n=36  EN only post-surgery (standard care)  Age, gender, weight, BMI matched to intervention group | 1536 (±425) | - | - |
| **OESOPHAGEAL CANCER: NO COMPARATOR GROUP** | | | | | | | | | |
| Becker Veronese *et al*. (2013) | 22.4 (±4.2) | Once, prior to starting treatment | 1422 (±348) | - | - | - | - | - | - |
|  |  |  |  |  |  |  |  |  |  |
| Haffejee *et al*. (1985) | Weight (kg)  45.2 (range 29-58) | Once, pre-surgery | 1028 (range 789-1099) | - | - | - | - | - | - |
| Khan *et al*. (2003)^4^ | - | Baseline, with varied prior palliative interventions | - | - | - | - | - | - | - |
|  |  | 2-weeks (post isocaloric diet period) | - | Mean change from baseline  -0.34 (95% CI  -0.95, 0.27)*^ns^* | - | - | - | - | - |
|  |  | 4-weeks (post 2 weeks of isocaloric diet plus daily thalidomide, 200mg) |  | Mean change from baseline  1.75 (95% CI  -0.42, 3.91)*^ns^* | - | - | - | - | - |
| Kobayashi *et al*. (2013) | - | Baseline (pre-surgery | - | - | - | - | - | - | - |
|  |  | POD 1, 2, 3, 4, 7, 14 | “POD 1-2 approximately 1550; POD 3-14 1600” | - | - | - | - | - | - |
| Kudo *et al*. (2022) | 22.1 (16.2-30.4)^5^ | Baseline (day of ESD procedure) | 1195 (608-1584)^5^ | 20.2 (IQR approximately 13, 26)^5,6^ | - | - | - | - | - |
|  |  |  |  |  |  |  |  |  |  |
|  |  | POD 1 | 1340 (848-2111)^5^  Higher than baseline (p<0.05) | 23.0 (IQR approximately 13, 32)^5,6^  Higher than baseline (p<0.05) |  |  |  |  |  |
| Legaspi *et al*. (1987) | 23.6 (±5.9) | Once, prior to starting treatment | - | 26.3 (±7.31) | - | - | - | - | - |
| Omagari *et al*. (2012) | 21.8 (range 19.8-24.6)^7^ | Once, timing unclear | 1225 (range 999-1770)^7^ | 22.2 (range 19.0-29.7)^7^ | - | - | - | - | - |
| Sato *et al*. (1997) | Weight (kg)  56.2 (range 45-80) | Baseline (pre-surgery | - | Results in Figure 1 | - | - | - | - | - |
|  |  | POD 1, 3, 5, 7 |  | Approximately 22kcal/kg/day at baseline, significant increase to 28-30kcal/kg/day from POD 1-7 (p<0.05) |  |  |  |  |  |
| Shinsyu *et al*. (2020) | 20.4 (±3.0) | Once, before or at least 30 days since receiving treatment | 1311 (±222) | 23.5 (±3.4) | 29.0 (±2.4)^1,8^ | - | - | - | - |
| Tashiro *et al*. (1999) | Weight (kg)  55.7 (±5.7) | Baseline (pre-surgery) | 1490 (±227) | 27.3 (±4.8) | - | - | - | - | - |
|  |  | POD 3 | 1883 (±297) | 33.7 (±1.8) |  |  |  |  |  |
| Yatabe *et al*. (2014) | 21 (±4) | Baseline (ICU admission post-surgery) | 1058 (±185) | 19.3 (±2.8) | - | - | - | - | - |
|  |  | Average during ventilation period (measures every 15 minutes) | 985 (±167) | 18.1 (±3.4) |  |  |  |  |  |
| GASTRIC CANCER: COMPARISON TO NON-CANCER CONTROL | | | | | | | | | |
| Cao *et al*. (2010) | 22.34 (3.3) | Once, prior to starting treatment | 1474 (±159)*^ns^* | - | 31.57 (±4.60)^1^  **Higher than non-cancer controls (p<0.05)** | Non-cancer control n=642  Age and gender matched  Weight and BMI higher than cancer group (p < 0.05) | 1448 (±188) | - | 30.31 (±4.07) |
| Rabito *et al*. (2013)^2^ | 20 (±3) | Once, pre-surgery | 1378 (±227)*^ns^* | 26 (±3)*^ns^* | - | Non-cancer control n=12  Majority female (66%)  Younger than cancer group (p<0.05)  Weight and BMI higher than cancer group (both p < 0.05) | 1588 (±210) | 26 (±3) | - |
| Sukkar *et al*. (2003) | 19.8 (±3.4) | Once, pre-surgery, at least 30 days since chemo/radiotherapy | 1353 (±210)*^ns^* | 25.2 (±3.4)  **Higher than non-cancer controls (p=0.002)** | - | Non-cancer control n=18  Age and gender matched  Weight and BMI higher than cancer group (p=0.001 and p=0.001, respectively) | 1456 (±157) | 21.7 (±2.4) | - |
| Yoshikawa *et al*. (2001) | Not reported | Once, timing unclear | 1255 (±119)*^ns^* | - | - | Non-cancer controls, n=6  No details of age, gender, weight, or BMI for either group, to allow comparison | 1323 (±166) | - | - |
| **GASTRIC CANCER: BETWEEN-GROUP COMPARISONS** | | | | | | | | | |
| Adachi *et al*. (2010) | Intervention group (n=10), IV ghrelin (3µ/kg, twice daily for 10 days  23.1 (±3.1) | Baseline (pre-surgery) | - | 22.6 (±6.1) | - | Gastric cancer in control arm of trial (n=10), IV saline placebo, twice daily for 10 days)  Age, gender, weight, BMI matched to intervention group | - | 21.8 (±4.0) | - |
|  |  | POD 10 |  | 21.4 (±6.0)  No significant change from baseline |  |  |  | 19.4 (±3.4)  Lower than baseline (p=0.023) |  |
| Hansell *et al*. (1987) | Intervention group 1 (n=10)  IV stanozol pre-surgery (single dose)  Weight (kg)  54.2 (SEM 4.8)  Intervention group 2 (n=10)  IV stanozol pre-surgery (single dose) plus twice daily IV naftidrofuryl from POD 0-4  Weight (kg)  59.5 (SEM 4.8) | Baseline (pre-surgery) | Data presented in Figure 2  Baseline mREE approximately 1250-1400kcal/day for both intervention groups | - | - | Gastric cancer patients in control arm of trial (n=10)  No additional IV infusions given  Age, gender, and weight matched to both intervention groups | Data presented in Figure 2  Baseline mREE approximately 1250-1400kcal/day for control group, similar to intervention groups |  |  |
|  |  | POD 2 | mREE (kcal/day) increased from baseline for both intervention groups. For Intervention 2 group this increase was statistically significant (p<0.05) |  |  |  | mREE (kcal/day) increased from baseline, non-significant  No significant difference in mREE (kcal/day) between control and intervention groups at POD 2 |  |  |
|  |  | POD 4 | mREE (kcal/day) decreased from POD 2 but remained higher than baseline for both intervention groups, changes not statistically significant |  |  |  | mREE (kcal/day) decreased from POD 2 but remained higher than baseline, non-significant  No significant difference in mREE (kcal/day) between control and intervention groups at POD 4 |  |  |
| Liu *et al*. (2012) | Intervention group (n=32)  Intensive, continuous insulin therapy post surgery  23.83 (±1.37) | Baseline (pre-surgery) | 1390 (±172) | Data in Figure 2C  Approximately 22.0 kcal/kg/day | - | Gastric cancer patients, in control arm of trial (n=32)  Standard insulin therapy post surgery  Age, gender, weight, BMI matched | Data in Figure 2A  Less than intervention group, approximately 1370kcal/day | Data in Figure 2C  Approximately 22.0 kcal/kg/day | - |
|  |  | POD 1 | 22.15% higher than baseline (p<0.05) | 27.22 (±1.33)  Lower than control group (p=0.0008) | - |  | 26.28% higher than baseline (significance not reported) | 29.97 (±1.47) | - |
|  |  | POD 3 | 11.07% higher than baseline (p<0.05) | 24.72 (±1.43)  Lower than control group (p=0.013) | - |  | 17.79% higher than baseline (significance not reported) | 25.66 (±1.63) | - |
|  |  | POD 7 | 11.05% higher than baseline (p<0.05) | Data in Figure 2C  Approximately 25.0 kcal/kg/day | - |  | 15.30% higher than baseline (significance not reported) | Data in Figure 2C  Approximately 25.75 kcal/kg/day | - |
| Wang *et al*. (2010) | Intervention group (n=45)  ‘Fast-track’ care post gastrectomy  23.85 (±2.4) | Baseline (pre-surgery) | 1181 (±132)*^ns^* | - | - | Gastric cancer patients, in control arm of trial (n=47)  Conventional care post gastrectomy  Age, gender, weight and BMI matched to intervention group | 1196 (±118) |  |  |
|  |  | POD 1 | 1370 (±147)  Lower than control group at POD 1 (p<0.05) |  |  |  | 1469 (±152) |  |  |
|  |  | POD 3 | 1264 (±154)  Lower than control group at POD 3 (p<0.05) |  |  |  | 1346 (±155) |  |  |
|  |  | POD 7 | 1199 (±110)*^ns^* |  |  |  | 1209 (±125) |  |  |
| **GASTRIC CANCER: NO COMPARATOR GROUP** | | | | | | | | | |
| Barcellos *et al*. (2021) | Not reported by cancer type | Once, prior to starting treatment | 1993 (±513) | 31.2 (±9.2) | - | - | - | - |  |
| Chinda *et al*. (2017) | Weight (kg)  60.0 (±13.0) | Baseline (pre-ESD procedure) | 1170 (±209) | 20.2 (±3.0) | - | - | - | - | - |
|  |  |  |  |  |  |  |  |  |  |
|  |  | POD 1 | 1238 (±236)  Higher than baseline (p<0.001) | 21.7 (±3.2)  Higher than baseline (p<0.001) |  |  |  |  |  |
| Hansell *et al*. (1986) | Weight (kg)  55.7 (SEM 3.2) | Once, timing unclear | 1266 (SEM 45) | 23.3 (SEM 0.7) | 27.7 (SEM 0.8)^9^ | - | - | - | - |
| Legaspi *et al*. (1987) | 19.19 | Once, prior to starting treatment | - | 24.9 | - | - | - | - |  |
| Omagari *et al*. (2012) | 19.0 (16.5-30.6)^7^ | Once, timing unclear | 1212 (932-1926)^6^ | 24.8 (19.0-29.4)^7^  Higher than patients in this study with liver cancer (p<0.05) | - | - | - | - | - |
| Shinsyu *et al*. (2020) | 22.7 (±3.3) | Once, before or at least 30 days since receiving treatment | 1319 (±227) | 21.9 (±3.2) | 30.7 (±4.8)^1,8^ | - | - | - | - |
| PANCREATIC CANCER: COMPARISON TO NON-CANCER CONTROL | | | | | | | | | |
| Barber *et al*. (2000) | Weight (kg)  55.2 (48.8, 61.2)^5^ | Baseline (at diagnosis) | 1360 (1170-1451)^5,^ *^ns^* | 24.0 (22.3, 27.0)^5^  **Higher than non-cancer controls (p<0.005)** | 33.8 (28.9, 35.3)^1,5^  **Higher than non-cancer controls (p<0.05)** | Non-cancer controls, n=6  Gender matched  Younger (p<0.05), with higher weight (p<0.01) than cancer group | 1475 (1270, 1750)^5^ | 18.8 (17.6, 21.1)^5^ | 28.7 (26.4, 29.8)^5^ |
|  |  | 3 weeks (post intervention, twice daily fish-oil-enriched oral supplement) | 1300 (1190, 1470)^5^  No significant change from baseline | 23.6 (20.2, 25.8)^5^  Lower than baseline (p<0.05) | 31.8 (27.7, 33.9)^1,5^  Lower than baseline (p<0.05) | - | - | - | - |
| Barber *et al*. (2004) | 20.4 (16.4-22.9)^7^ | Once, at diagnosis | 1425 (1035-1710)^7,^ *^ns^* | 24.3 (19.5-27.7)^7^  **Higher than non-cancer controls (p=0.046)** | - | Non-cancer controls, n=6  Gender matched  Younger (p=0.018), with higher BMI (p=0.01) than cancer group | 1565 (1270-1940)^7^ | 20.2 (16.3-23.3)^7^ | - |
| Cao *et al*. (2010) | 22.27 (±2.63) | Once, prior to starting treatment | 1479 (±168)*^ns^* | - | 31.66 (±4.09)^1^  **Higher than non-cancer controls p<0.05)** | Non-cancer controls n=642  Age and gender matched  Weight and BMI higher than cancer group (p<0.05) | 1448 (±188) | - | 30.31 (±4.07) |
| De Jong *et al*. (2005) | 22.6 (±1.0) | Once, pre-surgery | - | 23.8 (±1.5)*^ns^* | - | Non-cancer controls, n=11  Age and gender matched  Weight and BMI higher than cancer group (p<0.05) | - | 21.8 (±1.2) | - |
| Falconer *et al*. (1994) | Weight (kg)  59.3 (SEM 2.8) | Once, at diagnosis | - | 25.9 (SEM 1.2)  **Higher than non-cancer controls (p=0.0001)** | 31.5 (SEM 1.7)^1^  **Higher than non-cancer controls (p=0.0023)** | Non-cancer controls n=16  Age and gender matched  Weight higher than cancer group (p=0.0064) | - | 19.4 (SEM 0.7) | 24.6 (SEM 1.0) |
| Vaisman *et al*. (2012) | Post-surgical group n=15  18.85 (SEM 1.37) | Once, timing unclear for non-surgical group, or 1-6 months post-surgery | 1385 (SEM 80)  **Lower than non-cancer controls** **(p<0.001)** | - | 35.7 (SEM 1.1)^10,^*^ns^* | Non-cancer controls, n=75  BMI higher than both cancer groups (p<0.0001)  Age, gender, weight comparisons not reported | 1689 (SEM 38) | - | 33.3 (SEM 0.5)^10^ |
|  | Non-surgical group n=30  21.36 (SEM 1.03) |  | 1378 (SEM 61)  **Lower than non-cancer controls (p<0.001)** |  | 34.4 (SEM 0.8)^10,^*^ns^* |  |  |  |  |
| Wigmore *et al*. (1995) | Weight (kg)  58.6 (SEM 3.8) | Once, timing unclear for baseline assessment compared to non-cancer controls | 1499 (SEM 71)  **Higher than non-cancer controls (p<0.02)** | 25.58 (SEM 1.2)  **Higher than non-cancer controls (p<0.001)** | 35.0 (SEM 0.9)^1^  **Higher than non-cancer controls (p<0.001)** | Non-cancer controls, n=17  Age and gender matched  Weight higher than cancer groups (p<0.05) | 1377 (SEM 58) | 19.15 (SEM 0.7) | 26.2 (SEM 0.5) |
| **PANCREATIC CANCER: BETWEEN-GROUP COMPARISONS** | | | | | | | | | |
| Moses *et al*. (2004) | 20 (SEM 1) | Baseline, timing unclear | 1387 (SEM 42)  TEE: 1732 (SEM 82) | - | - | - | - | - | - |
|  |  | 8 weeks  Subgroup receiving intervention, n=7  (daily oral supplements with added EPA) | Mean change from baseline  -1 (SEM 42) (p>0.05)*^ns^*  TEE: 286 (SEM 79) Higher than baseline (p<0.05)*^ns^* |  |  | Pancreatic cancer, in control arm of trial, n=12 (daily oral supplements with no added EPA)  Age, gender, BMI matched at baseline | Mean change from baseline  -15 (SEM 25) (p>0.05)  TEE: 99 (SEM 132) (p>0.05) | - | - |
| Wigmore *et al*. 1995 | Intervention group (n=10)  Weight (kg)  58.6 (SEM 3.8) | Baseline, timing unclear | 1468 (SEM 99) | 25.62 (SEM 0.9) | 35.18 (SEM 1.0) | Pancreatic cancer, in control arm of trial, n=6 (daily placebo for 7 days)  Age, gender, weight matched at baseline | 1518 (SEM 61) | 25.55 (SEM 0.4) | 34.83 (SEM 2.6) |
|  |  | 7 days (post intervention, daily ibuprofen (1200mg)) | 1386 (SEM 89)  Lower than baseline (p<0.02) | 24.53 (SEM 0.6)  Lower than baseline (p<0.05) | 33.12 (SEM2.4)  Lower than baseline (p<0.002) |  | 1564 (SEM 77)  No significant change from baseline (p>0.05) | 26.02 (SEM 0.7)  No significant change from baseline (p>0.05) | 35.22 (SEM 2.4)  No significant change from baseline (p>0.05) |
| Wigmore *et al*. (1997) | High CRP (≥10mg/L) group (n=19)  Weight (kg)  60 (53-66)^5^ | Once, with varied prior palliative interventions | - | 26.6 (25.0-29.9)^5^  Higher than normal CRP group (p<0.002) | - | Pancreatic cancer with normal CRP levels (<10mg/L), n=16  Similar age and weight to group with high CRP (p>0.05)  Gender comparison not reported | - | 23.3 (19.8-25.2)^5^ | - |
| **PANCREATIC CANCER: NO COMPARATOR GROUP** | | | | | | | | | |
| Barber *et al*. (1999) | 19.8 (17.8, 21.8)^5^ | Baseline (at diagnosis) | 1339 (1159, 1148)^5^ | 24.2 (23.1, 27.7)^5^ | 34.0 (29.6, 35.6)^1,5^ | - | - | - | - |
|  |  |  |  |  |  |  |  |  |  |
|  |  | 3 weeks (post intervention, twice daily fish-oil-enriched oral supplement) | 1303 (1186, 1470)^5^  No significant change from baseline | 24.0 (20.2, 25.8)^5^  Lower than baseline (p=0.025) | 31.8 (27.7, 33.9)^1,5^  Lower than baseline (p=0.018) |  |  |  |  |
| Barcellos *et al*. (2021) | Not reported by cancer type | Once, prior to starting treatment | 1747 (± 750) | 29.0 (± 10.7) | - | - | - | - | - |
| Kim *et al*. (2013) | 27 | Baseline (at diagnosis) | 1707 | 24.4 | - | - | - | - | - |
|  |  | 2 months follow up | 2416 | 35 | - | - | - | - | - |
| Omagari *et al*. (2012) | 20.5 (range 18.8-25.3)^7^ | Once, timing unclear | 1422 (range 969-1592)^7^ | 23.7 (range 20.7-27.8)^7^ | - | - | - | - | - |
| Terepka *et al*. (1956) | Weight (kg)  52.55 | Baseline, timing unclear (days 0-12) | 1850 (average of 12 consecutive daily measures) | - | - | - | - | - | - |
|  |  | Period of ‘forced feeding (days 13-24) | Results in Figure 1  Slight increase during period of ‘forced feeding’ (non-significant) |  |  |  |  |  |  |
| Wigmore *et al*. (1996) | Weight (kg)  62 (52-70)^5^ | Baseline, with varied prior palliative interventions | - | 25 (21-27)^5^ | - | - | - | - | - |
|  |  | 1 month post commencement of fish oil intervention, starting at 2g/day, increased weekly by 2g to maximum 16g/day |  | 24 (20-27)^5^  No significant change from baseline (p-value not reported) |  |  |  |  |  |
|  |  | Additional follow up (median 3 months, IQR 3-5 months) |  | 26 (23-28)^5^  No significant change from baseline (p-value not reported) |  |  |  |  |  |
| **BILE DUCT CANCER: NO COMPARATOR GROUP** | | | | | | | | | |
| Barcellos *et al*. (2021) | Not reported by cancer type | Once, prior to starting treatment | 1994 (±807) | 30.7 (±12.3) | - | - | - | - | - |
| Omagari *et al*. (2012) | 20.5 (range 18.8-25.3)^7^ | Once, timing unclear | 1422 (range 969-1592)^7^ | 23.7 (range 20.7-27.8)^7^ | - | - | - | - | - |
| **LIVER CANCER: COMPARISON TO NON-CANCER CONTROL** | | | | | | | | | |
| Chen *et al*. (1994) | Weight (kg)  65.2 (±1.4) | Once, pre-surgery | 1433 (SEM 33)  **Higher than non-cancer controls (p<0.005)** | 22.12 (SEM 0.36)  **Higher than non-cancer controls (p<0.05)** | - | Non-cancer controls, n=60  Gender matched, similar age and weight to cancer group | 1320 (SEM 19) | 20.99 (SEM 0.27) | - |
| Guglielmi *et al*. (1992) | Well-nourished (n=8)  26 (±4) | Once, timing unclear | 1701 (±151)  **Higher than non-cancer controls (p<0.02)** | - | 33.1 (±4.5)^1,^ *^ns^* | Non-cancer controls, n=6  Age and gender matched  Similar weight and BMI to cancer group | 1491 (±219) | - | 30.0 (±3.9) |
|  | Malnourished (n=5)  24 (± 2) |  | 1854 (± 313)  **Higher than non-cancer controls (p<0.02)** |  | 31.8 (±1.6)^1,^ *^ns^* |  |  |  |  |
| Henz *et al*. (2021) | 27.0 (±4.0) | Once, timing unclear | 1643 (±364)*^ns^* | - | - | Non-cancer controls, n=85  More females, and younger age than cancer group (both p=0.001)  Similar weight and BMI to cancer group | 1526 (±277) | - | - |
| Merli *et al*. (1992) | 24.8 (±2.5) | Once, timing unclear | 1551 (±154)  **Higher than non-cancer controls (p-value not reported)** | 24.7 (±2.7)  **Higher than non-cancer controls (p<0.005)** | 34.3 (±3.36)^3^  **Higher than non-cancer controls (p<0.005)** | Non-cancer controls, n=12  Gender matched, ‘comparable’ weight and BMI to cancer group (p-values not reported)  Younger than cancer group (p<0.01) | 1350 (±232) | 21.7 (±1.8) | 28.9 (±3.7)^3,11^ |
| Ren *et al*. (2019) | 22.63 (±3.83) | Once, timing unclear | 1396 (±333)*^ns^* | - | - | Non-cancer controls, n=75  Similar age (p=0.081), gender (p=0.169), and BMI (p=0.096) to cancer group | 1468.4 (±355) | - | - |
| **LIVER CANCER: BETWEEN-GROUP COMPARISONS** | | | | | | | | | |
| Saito *et al*. (2013) | One-year mortality post TACE (n=22)  24.4 (±2.6) | Baseline (pre-TACE) | 1284 (±177)*^ns^* | - | - | Liver cancer, survived ≥ one year post TACE, n=78  Similar age (p=0.436), gender (p=0.621), weight (p=0.468) and BMI (p=0.096) to one-year mortality group | 1348 (±229) | - | - |
|  |  | 7 days post TACE | Factor ratio (post TACE ÷ pre-TACE)  1.01 (range 0.88-1.12)^7,^ *^ns^* |  |  |  | Factor ratio (post TACE ÷ pre-TACE)  0.97 (range 0.78-1.22)^7^ |  |  |
| Saito *et al*. (2014) | Intervention group (n=13)  23.6 (±0.9) | Baseline (pre-RFA) | 1312 (±62)*^ns^* | - | - | Liver cancer in control arm of trial, n=27  Similar age (p=0.144), gender (p=1.000), weight (p=0.456) and BMI (p=0.441) to intervention group | 1390 (±45) |  |  |
|  |  | 7 days post RFA | Factor ratio (post-RFA ÷ pre-RFA)  0.97 (±0.02)*^ns^* |  |  |  | Factor ratio (post-RFA ÷ pre-RFA)  0.97 (±0.02) |  |  |
| **LIVER CANCER: NO COMPARATOR GROUP** | | | | | | | | | |
| Barcellos *et al*. (2021) | Not reported by cancer type | Once, prior to starting treatment | 1795 (±772) | 25.1 (±10.9) | - | - | - | - | - |
| Omagari *et al*. (2012) | 24.3 (range 16.7-29.4)^7^ | Once, timing unclear | 1275 (range 825-1897)^6^ | 20.6 (range 16.5-26.0)^7^ | - | - | - | - | - |
|  |  |  |  |  |  |  |  |  |  |

*^ns^*not significantly different from control/comparator group (*p* > 0.05); dash (-) indicates data not reported; AA amino acids; CI confidence interval; EE energy expenditure; EN enteral nutrition; EPA eicosapentaenoic acid; ESD endoscopic submuscosal dissection; F females; FFM fat-free mass; ICU intensive care unit; IQR interquartile range; M males; mREE measured resting energy expenditure; PN parenteral nutrition; POD post-operative day; pREE predicted resting energy expenditure; RFA radiofrequency ablation; TACE transcatheter arterial chemoembolization; TPN total parenteral nutrition; ^1^FFM measured using bioelectrical impedence analysis; ^2^oesophageal and gastric cancer data reported as one cohort, n = 24; ^3^FFM calculated using triceps skinfold thickness method (1); ^4^n=9, as one participant had missing data for energy expenditure; ^5^data reported as median (interquartile range); ^6^interquartile range data reported in figure 2; ^7^data reported as median (range); ^8^study has missing data for FFM (total cohort n=51, FFM data for n=46), however does not report cancer type/s of patients with this missing data (study included patients with oesophageal, gastric, and colorectal cancers); ^9^FFM derived from assessment of total body water using isotope dilution; ^10^FFM measured using dual-energy X-ray absorptiometry (DEXA); ^11^FFM data missing for one patient in control group, n=11

Additional Reference:

1. Durnin JV, Womersley J. Body fat assessed from total body density and its estimation from skinfold thickness: measurements on 481 men and women aged from 16 to 72 years. Br J Nutr. 1974;32(1):77-97.
