# Supplementary material for: Energy Expenditure in Upper Gastrointestinal Cancers: a Scoping Review
Source: Adv Nutr. 2023 Aug 8;14(6):1307–25. doi: 10.1016/j.advnut.2023.08.002 (PMC10721480; doi:10.1016/j.advnut.2023.08.002)
Supplement: Multimedia component3 [file mmc3.docx]

**Supplementary File 3**. Energy expenditure compared to predictive equations, by cancer type

| **Author, year** | **BMI (kg/m^2^)**  (mean ±SD) | **Timing of EE assessments** | **mREE**  (kcal/day) | **Predictive equation** | **pREE**  (kcal/day) | **mREE/pREE (%)**  (mean ±SD) | **Comparison to equation** |
| --- | --- | --- | --- | --- | --- | --- | --- |
| **OESOPHAGEAL CANCER** | | | | | | | |
| Becker Veronese *et al*. (2013) | 22.4 (4.2) | Once, prior to starting treatment | 1422 (±348) | Harris Benedict | 1311 (±215) | - | mREE significantly higher than pREE (p=0.014)  mREE was within ±10% of pREE for 26.7% of patients  n=6 (20%) hypo-, n=7 (23.3%) normo-, n=17 (56.7%) hypermetabolic^1^ |
| Boudou-Rouquette *et al*. (2022) | Not reported by cancer type | Once, prior to starting treatment | Not reported by cancer type | Harris Benedict | Not reported by cancer type | Not reported by cancer type | n=0 (0%) hypo-, n=2 (50%) normo-, n=2 (50%) hypermetabolic^1^ |
| Cao *et al*. (2010) | 22.53 (± 3.0) | Once, prior to starting treatment | 1480 (±147) | Harris Benedict | - | 109.44 (±13.15) | n=11 (7.4%) hypo-, n=65 (43.3%) normo-, n=74 (49.3%) hypermetabolic^1^ |
| Dempsey *et al*. (1984) | - | Once, timing unclear | Not reported by cancer type | Harris Benedict | Not reported by cancer type | Not reported by cancer type | n=9 (35%) hypo-, n=11 (42%) normo-, n=6 (23%) hypermetabolic^1^ |
| Hioki *et al*. (1990) | Weight (kg)  50 (±12) | Baseline (pre-surgery) | 1175 (±59) | Harris Benedict | 1112 (±71) | 106 (±5) | mREE/pREE significantly higher in this cohort than patients in this study with gastric cancer, on POD 1-3 (all p<0.001) |
|  |  | POD 1 | - |  |  | 144 (±16) |  |
|  |  | POD 2 | - |  |  | 137 (±7) |  |
|  |  | POD 3 | - |  |  | 135 (±5) |  |
|  |  | POD 4 | - |  |  | 134 (±6) |  |
|  |  | POD 5 | - |  |  | 129 (±6) |  |
| Klein *et al*. (1990) | Weight (kg)  58 (SEM 4) | Once, prior to starting treatment | - | Harris Benedict (weight adjusted) | 23.4 kcal/kg/day (SEM 0.3) | 94 (SEM 0.3) | No significant difference |
| Kudo *et al*. (2022) | 22.1 (16.2, 30.4)^2^ | Baseline (day of ESD procedure) | 1195 (608, 1584)^2^ | Harris Benedict | 1235 (941, 1677)^2^ | 95 (IQR approximately 0.79, 1.15)^2,3^ | - |
|  |  | POD 1 | 1340 (848, 2111)^2^ |  | 1247 (984, 1563)^2^ | 106 (IQR approximately 0.69, 1.42)^2,3^  Significantly higher than baseline (p<0.05) |  |
| Legaspi *et al*. (1987) | 23.6 (± 5.9) | Once, prior to starting treatment | - | Harris Benedict | - | 124.3 (±28.4) | - |
| Mitamura *et al*. (2011) | - | Once, prior to starting treatment | 1430 (±401) | Harris Benedict | 1218 (±111) | 120 (±30) | - |
| Okamoto *et al*. (2001) | 19.3 (± 1.9) | Baseline (pre-surgery) | 1257 (±110) | Harris Benedict | 1256 (±149) | 101 (±9) | Positive correlation between mREE and pREE (p<0.05) |
|  |  | POD 7 |  |  |  | 117 (±15)  Significantly higher than baseline (p<0.05) |  |
|  |  | POD 14 |  |  |  | 105 (±22)  Not significantly difference to baseline (p>0.05) |  |
| Omagari *et al*. (2012) | 21.8 (19.8-24.6)^4^ | Once, timing unclear | 1225 (999-1770)^4^ | Harris Benedict | - | 103 (90-137)^4^ | - |
| Satoh *et al*. (2018) | Intervention group 1 (n=10)  IV glucose 3g/hr with AA 1.2g/hr during surgery  22 (± 3)  Intervention group 2 (n=12)  IV glucose 4.5g/hr with AA 1.8g/hr during surgery  20 (± 3)  Control group (n=10)  IV therapy without glucose or AA during surgery  22 (± 4) | Baseline (during surgery) | 1230 (±228)  1317 (±282)  1012 (±153) | Harris Benedict | Intervention group 1  1275 (±174)  Intervention group 2  1248 (±176)  Control group  1142 (±192) | - | Control group mREE significantly lower than pREE (p<0.05) |
| Shinsyu *et al*. (2020) | 20.4 (± 3.0) | Once, before or at least 30 days since receiving treatment | 1311 (±222) | Harris Benedict | 1249 (±198) | 105 (±10) | - |
| Wu *et al*. (2013) | 21.6 (± 2.61) | Once, prior to starting treatment | 1595 (±325) | Harris Benedict | 1321 (±167) | 121.2 (±22.91) | n = 38 (67.9%) were hypermetabolic^1^ |
| Yatabe *et al*. (2014) | 21 (± 4) | Baseline (post-op ICU admission) | 1058 (±185) | Harris Benedict | 1191 (±159) | 89 (±9) | mREE significantly lower than equation (p<0.001) |
|  |  | Average during ventilation period (measures every 15-minutes) | 985 (±167) |  |  | 83 (±10) | mREE significantly lower than equation (p<0.001) |
| **GASTRIC CANCER** | | | | | | | |
| Cao *et al*. (2010) | 22.34 (3.3) | Once, prior to starting treatment | 1474 (±159) | Harris Benedict | - | 108.72% (±13.60) | n=13 (8.4%) were hypo-, n=66 (42.9%) were normo-, and n=75 (48.7%) were hypermetabolic^1^ |
| Boudou-Rouquette *et al*. (2022) | Not reported by cancer type | Once, prior to starting treatment | Not reported by cancer type | Harris Benedict | Not reported by cancer type | Not reported by cancer type | n=0 (0%) hypo-, n=2 (50%) normo-, n=2 (50%) hypermetabolic^1^ |
| Chinda *et al*. (2017) | Weight (kg)  60.0 (±13.0) | Baseline (pre-ESD procedure) | 1170 (±209) | Harris Benedict | - | 96 (±11) | - |
|  |  |  |  |  |  |  |  |
|  |  | POD 1 | 1238 (±236) |  |  | 103 (±14)  Significantly higher than baseline (p<0.001) |  |
| Dempsey *et al*. (1984) | Not reported by cancer type | Once, timing unclear | Not reported | Harris Benedict | Not reported by cancer type | - | n=9 (32%) hypo-, n=8 (29%) normo-, n=11 (39%) hypermetabolic^1^ |
| Hansell *et al*. (1986) | Weight (kg)  55.7 (SEM 3.2) | Once, timing unclear | 1266 (SEM 45) | Harris Benedict | 1214 (SEM 44) | - | No significant difference |
| Hioki *et al*. (1990) | Weight (kg)  48 (±8) | Baseline (pre-surgery) | 1156 (±207) | Harris Benedict | 1152 (±170) | 100 (±11) | mREE/pREE significantly lower in this cohort than patients in this study with oesophageal cancer, on POD 1-3 (all p<0.001) |
|  |  | POD 1 | - |  |  | 113 (±10) |  |
|  |  | POD 2 | - |  |  | 124 (±6) |  |
|  |  | POD 3 | - |  |  | 123 (±4) |  |
|  |  | POD 4 | - |  |  | 132 (±6) |  |
|  |  | POD 5 | - |  |  | 131 (±6) |  |
| Ishikawa *et al*. (2004) | - | Baseline (pre-surgery) | - | Harris Benedict | - | Baseline  119 (±18) | Data presented in Figure 1.  Slight increase in mREE/pREE from baseline to POD 1, then gradual decrease to approximately 115% (below baseline value) by POD 14. No significant differences in mREE/pREE between assessment time points (all p>0.05) |
|  |  | POD 1, 3, 7, 14 |  |  |  |  |  |
| Legaspi *et al*. (1987) | 19.19 | Once, prior to starting treatment | - | Harris Benedict | - | 122 | - |
| Omagari *et al*. (2012) | 19.0 (16.5-30.6)^4^ | Once, timing unclear | 1212 (932-1926)^4^ | Harris Benedict | - | 105 (86-131)^4^ | - |
| Shinsyu *et al*. (2020) | 22.7 (± 3.3) | Once, before or at least 30 days since receiving treatment | 1319 (±227) | Harris Benedict | 1312 (±234) | 101 (±7) | - |
| Sukkar *et al*. (2003) | 19.8 (± 3.4) | Once, pre-surgery, at least 30 days since chemo/radiotherapy | 1353 (±210) | Harris Benedict | 1196 (±109) | mREE higher than pREE (p-value not reported) |  |
| Yoshikawa *et al*. (2001) | - | Once, timing unclear | 1255 (±119) | Harris Benedict | - | 102.8 (±9.3) | - |
| **PANCREATIC CANCER** | | | | | | | |
| Cao *et al*. (2010) | 22.27 (± 2.63 | Once, prior to starting treatment | 1479 (±168) | Harris Benedict | - | 112.0 (±13.4) | n=5 (3.9%) were hypo-, n=48 (37.5%) were normo-, and n=75 (58.6%) were hypermetabolic^1^ |
| Bauer *et al*. (2004) | 24.4 (± 3.3) | Once, during palliative treatment | 1568 (±270) | Harris Benedict | 1572 (±280) | - | No significant difference, p=0.924 |
|  |  |  |  | Schofield | 1631 (±307) |  | No significant difference, p=0.347 |
|  |  |  |  | Owen | 1582 (±262) |  | No significant difference, p=0.771 |
|  |  |  |  | Mifflin | 1513 (±297) |  | No significant difference, p=0.346 |
|  |  |  |  | Cunningham | 1531 (±235) |  | No significant difference, p=0.505 |
|  |  |  |  | Wang | 1562 (±234) |  | No significant difference, p=0.931 |
|  |  |  |  | 20kcal/kg ratio | 1513 (±318) |  | No significant difference, p=0.367 |
| Boudou-Rouquette *et al*. (2022) | Not reported by cancer type | Once, prior to starting treatment | - | Harris Benedict | Not reported by cancer type | Not reported by cancer type | n=2 (22%) hypo-, n=4 (44%) normo-, hypermetabolic data not reported^1^ |
| Dempsey *et al*. (1984) | Not reported by cancer type | Once, timing unclear | - | Harris Benedict | Not reported by cancer type | - | n=15 (54%) hypo-, n=9 (32%) normo-, n=4 (14%) hypermetabolic^1^ |
| Kim *et al*. (2013) | 27 | Baseline (at diagnosis) | 1707 | Harris Benedict | 1545 | 110.5 | - |
|  |  | 2 months | 2416 |  | 1531 | 157.8 | - |
| Moses *et al*. (2004) | 20 (SEM 1) | Baseline, timing unclear | 1387 (SEM 42)  TEE: 1732 (SEM 82) | Schofield  TEE: Schofield x 1.5 | 1268 (SEM 32)  TEE: 1903 (SEM 48) | - | mREE significantly higher than predicted (p = 0.001)  mTEE significantly lower than predicted (p = 0.023) |
| Omagari *et al*. (2012) | 20.5 (18.8-25.3)^4^ | Once, timing unclear | 1422 (969-1592)^4^ | Harris Benedict | - | 107 (97-118)^4^ | - |
| Vaisman *et al*. (2012) | Post-surgical group (n=15)  18.85 (SEM 1.37) | Once, timing unclear for non-surgical group, or 1-6 months post-surgery | 1385 (SEM 80) | Harris Benedict | - | 111.0 (SEM 3.2) | - |
|  | Non-surgical group (n=30)  21.36 (SEM 1.03) |  | 1378 (SEM 61) |  |  | 106.9 (SEM 2.4) |  |
| Witvliet-van Nierop *et al*. (2017) | 23.9 (± 2.5) | Once, at varied points of treatment | 1829 (1622-2030)^2^ | FAO/WHO/UNU | 1372 (1316-1440)^2^ | 133 (115-117)^2^ | - |
| **BILE DUCT CANCER** | | | | | | | |
| Dempsey *et al*. (1984) | Not reported by cancer type | Once, timing unclear | - | Harris Benedict | Not reported by cancer type | - | n=7 (78%) hypo-, n=1 (11%) normo-, and n=1 (11%) were hypermetabolic^1^ |
| Omagari *et al*. (2012) | 20.5 (18.8-25.3)^4^ | Once, timing unclear | 1422 (969-1592)^4^ | Harris Benedict | - | 107 (97-118)^4^ | - |
| **LIVER CANCER** | | | | | | | |
| Chen *et al*. (1994) | Weight (kg)  65.2 (SEM 1.4) | Once, pre-surgery | 1433 (SEM 33) | Harris Benedict | 1415 (SEM 27) | 101.5 (SEM 1.4) | - |
| Dempsey *et al*. (1984) | Not reported by cancer type | Once, timing unclear | - | Harris Benedict | Not reported by cancer type | - | n=7 (78%) hypo-, n=1 (11%) normo-, n=1 (11%) hypermetabolic^1^ |
| Guglielmi *et al*. (1992) | Well-nourished (n=8)  26 (± 4) | Once, timing unclear | 1701 (±151) | Harris Benedict | 1440 (±167) | - | mREE (kcal/day) significantly higher than pREE (p<0.01) |
|  | Malnourished (n=5)  24 (± 2) |  | 1854 (±313) |  | 1528 (±242) |  | No significant difference between mREE and pREE (p>0.05) |
| Henz *et al*. (2021) | 27.0 (± 4.0) | Once, timing unclear | 1643 (±364) | Harris Benedict | 1466 (±224) | - | mREE significantly higher, MD 177 (95% CI 9.8, 293), p=0.004 |
|  |  |  |  | Schofield | 1489 (±203) |  | mREE significantly higher, MD 154 (95% CI 36.9, 270), p=0.011 |
|  |  |  |  | WHO 2000 | 1518 (±208) |  | mREE significantly higher, MD 125 (95% CI 9.1, 240), p=0.035 |
|  |  |  |  | Mifflin | 1433 (±196) |  | mREE significantly higher, MD 210 (95% CI 98.5, 321), p=0.001 |
|  |  |  |  | FAO/WHO/UNU | 1522 (±208) |  | No significant difference, MD 120 (95% CI -2.3, 243), p=0.054 |
|  |  |  |  | IOM | 1402 (±168) |  | mREE significantly higher, MD 241 (95% CI 116, 366), p<0.001 |
|  |  |  |  | Cunningham | 1629 (±233) |  | No significant difference, MD 14 (95% CI -101, 129), p=0.806 |
|  |  |  |  | McArdle | 1478 (±229) |  | mREE significantly higher, MD 165 (95% CI 49.5, 279), p=0.006 |
| Merli *et al*. (1992) | 24.8 (± 2.5) | Once, timing unclear | 1551 (±154) | Harris Benedict | - | 120 (±10) | - |
| Omagari *et al*. (2012) | 24.3 (16.7-29.4)^4^ | Once, timing unclear | 1275 (825-1897)^4^ | Harris Benedict | - | 102 (84-119)^4^ | - |
| Ren *et al*. (2019) | 22.63 (± 3.83) | Once, timing unclear | 1396 (±332.62) | Harris Benedict | 1471 (±234) | 95.58 (±19.65) | - |
| Sugihara *et al*. (2014) | 22.3 (SEM 0.6) | Baseline (pre-surgery) | - | Harris Benedict | - | Approximately 100% | Results in Figure 2  No significant difference in mREE/pREE between any time points |
|  |  | POD 7 |  |  |  | Approximately 96% |  |
|  |  | POD 14 |  |  |  | Approximately 101% |  |

A dash (-) indicates data not reported; CI confidence interval; ESD endoscopic submuscosal dissection; IQR interquartile range; IOM Institute of Medicine; MD mean difference; mREE measured resting energy expenditure; mTEE measured total energy expenditure; pREE predicted resting energy expenditure; POD post-operative day; ^1^hypometabolic = mREE/pREE < 90%, normometabolic = mREE/pREE between 90-110%, hypermetabolic = mREE/pREE > 110%, as per Boothby *et al*; ^2^data reported as median (interquartile range); ^3^interquartile range data reported in Figure 2; ^4^data reported as median (range)
